# Inducible Synthetic Growth Regulation Using the ClpXP Proteasome Enhances cis,cis-Muconic Acid and Glycolic Acid Yields in *Saccharomyces cerevisiae*

**DOI:** 10.1021/acssynbio.2c00467

**Published:** 2023-03-28

**Authors:** Natalia Kakko, Anssi Rantasalo, Tino Koponen, Virve Vidgren, Matti Kannisto, Natalia Maiorova, Heli Nygren, Dominik Mojzita, Merja Penttilä, Paula Jouhten

**Affiliations:** † VTT Technical Research Centre of Finland Ltd, Espoo 02044 VTT, Finland; ‡ School of Chemical Engineering, Department of Bioproducts and Biosystems, Aalto University, P.O. Box 16300, Espoo FI-00076 AALTO, Finland

**Keywords:** synthetic regulation, Saccharomyces cerevisiae, ClpXP proteasome, cis,cis-muconic acid, glycolic acid

## Abstract

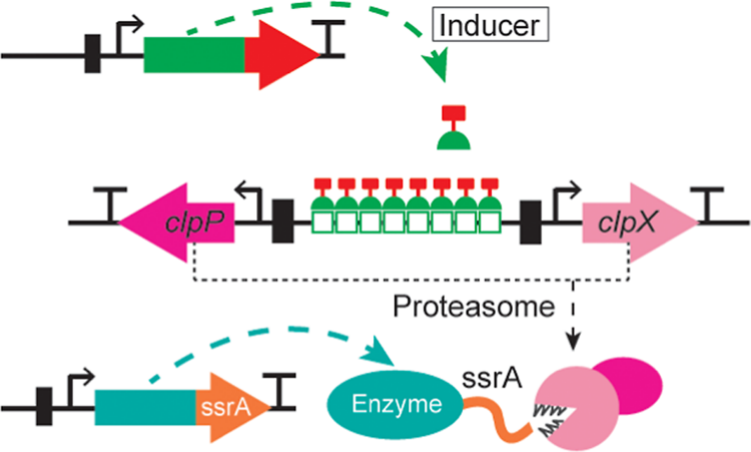

Engineered microbial cells can produce sustainable chemistry, but the production competes for resources with growth. Inducible synthetic control over the resource use would enable fast accumulation of sufficient biomass and then divert the resources to production. We developed inducible synthetic resource-use control over*Saccharomyces cerevisiae* by expressing a bacterial ClpXP proteasome from an inducible promoter. By individually targeting growth-essential metabolic enzymes Aro1, Hom3, and Acc1 to the ClpXP proteasome, cell growth could be efficiently repressed during cultivation. The ClpXP proteasome was specific to the target proteins, and there was no reduction in the targets when ClpXP was not induced. The inducible growth repression improved product yields from glucose (cis,cis-muconic acid) and per biomass (cis,cis-muconic acid and glycolic acid). The inducible ClpXP proteasome tackles uncertainties in strain optimization by enabling model-guided repression of competing, growth-essential, and metabolic enzymes. Most importantly, it allows improving production without compromising biomass accumulation when uninduced; therefore, it is expected to mitigate strain stability and low productivity challenges.

## Introduction

Engineered microbial cells are capable of synthesizing diverse chemical compounds from, e.g., bioplastic precursors to cannabinoids and insect pheromones.^1−4^ The synthesis is achieved by expressing a combination of genes from various origins within the host cells’ native metabolic pathways. Such assembled synthetic pathways require resources, i.e., precursors, energy, and redox power, from the native metabolism. For diverting sufficient resources to a synthetic pathway in order to reach industrially attractive production, the native pathways commonly need substantial optimization.^5,6^ However, the synthetic pathways are usually in resource competition with cell growth or processes linked to that (e.g., growth-essential byproduct formation), and simply knocking out such competing native pathways is not feasible. Therefore, the resource share of desired compound production has been increased by reducing competing activities,^7−9^ forcing biomass synthesis via alternative lower-yield routes,^10−12^ or by using product sensors to control growth-essential gene expression.^13^ While shown to be successful in demonstration cases, the product sensor-driven control options are limited to available sensors,^14,15^ and forcing growth to occur via alternative routes tends to require a high number of deletions.^16^ Most challengingly, when high product yields are achieved with these approaches, cell growth is inevitably compromised, leading to a loss of competitiveness against contaminants in the raw material or process, low productivities, and likely cell stability and viability issues.

To circumvent the issues following from the growth compromise, two-stage fermentations using dynamic regulation or inducers for controlling resource distribution in host cells have been proposed.^17^ Dynamic regulation or inducible systems would be used to change the cellular phenotype during the cultivation process. It could time-separate growth and production to first accumulate biomass, and in a second fermentation phase, to silence the growth-essential but production-competing pathways. Such switch in the cellular phenotype is a complex natural trait of some organisms, e.g., secondary metabolite producing bacteria and fungi in response to certain growth-essential nutrient limitation.^18,19^ It is, however, not simply transferable to other species, many of which commonly respond to nutrient limitation by reducing the metabolic activities to a bare minimum^20^ or even entering into quiescence.^21,22^ Thus, avoiding nutrient limitation using inducible synthetic regulation to repress metabolic pathways linked to growth appears appealing. In a common eukaryotic production host, yeast*Saccharomyces cerevisiae*, switches in cellular phenotypes during cultivation processes have been achieved by using inducible promoters^23−26^ or transcription factors,^27^ a heterologous quorum sensing system coupled to RNA interference for gene expression control,^28^ an auxin-responsive protein degradation system,^29^ and Clustered Regularly Interspaced Short Palindromic Repeats interference (CRISPRi) triggered from an inducible promoter.^30^ The approaches are conceptually promising for developing efficient producer strains and processes. For instance, the quorum sensing-coupled RNA interference and auxin-inducible degradation targeted to single growth-essential enzymes have been demonstrated to deliver substantially improved product titers (i.e., *para*-hydroxybenzoic acid titer by 41%^28^ and nerodiol titer by 36%^29^). However, these previous attempts have compromised the target activities already prior to the intended switch,^29,31^ relied upon induction not possible during glucose utilization,^30^ interfered with the native proteasome,^29^ or have been inefficient in counteracting native regulation.^32^

We demonstrate here a novel inducible synthetic regulation system in *S. cerevisiae* addressing the previous challenges. The regulation system is implemented using the ClpXP proteasome of bacterial origin previously established in yeast by Grilly et al. (2007)^33^ which we express from an inducible promoter for achieving control intervention during a production process. We show how the system allows efficient wild type growth of cells before the induction and enables switching the cellular phenotype and ceasing growth guided by first-principle metabolic modeling. We further demonstrate how the inducible synthetic regulation can be used to enhance the resource share of heterologous compound production. As model products, we use two industrially relevant platform chemicals cis,cis-muconic acid (MA) and glycolic acid (GA) and synthesize them using an *S. cerevisiae* background strain without native metabolic pathway optimization.

## Results and Discussion

### Introducing the ClpXP Proteasome under Tet-On Induction in *S. cerevisiae* Makes a Tunable Synthetic Regulator

**Figure 1:**
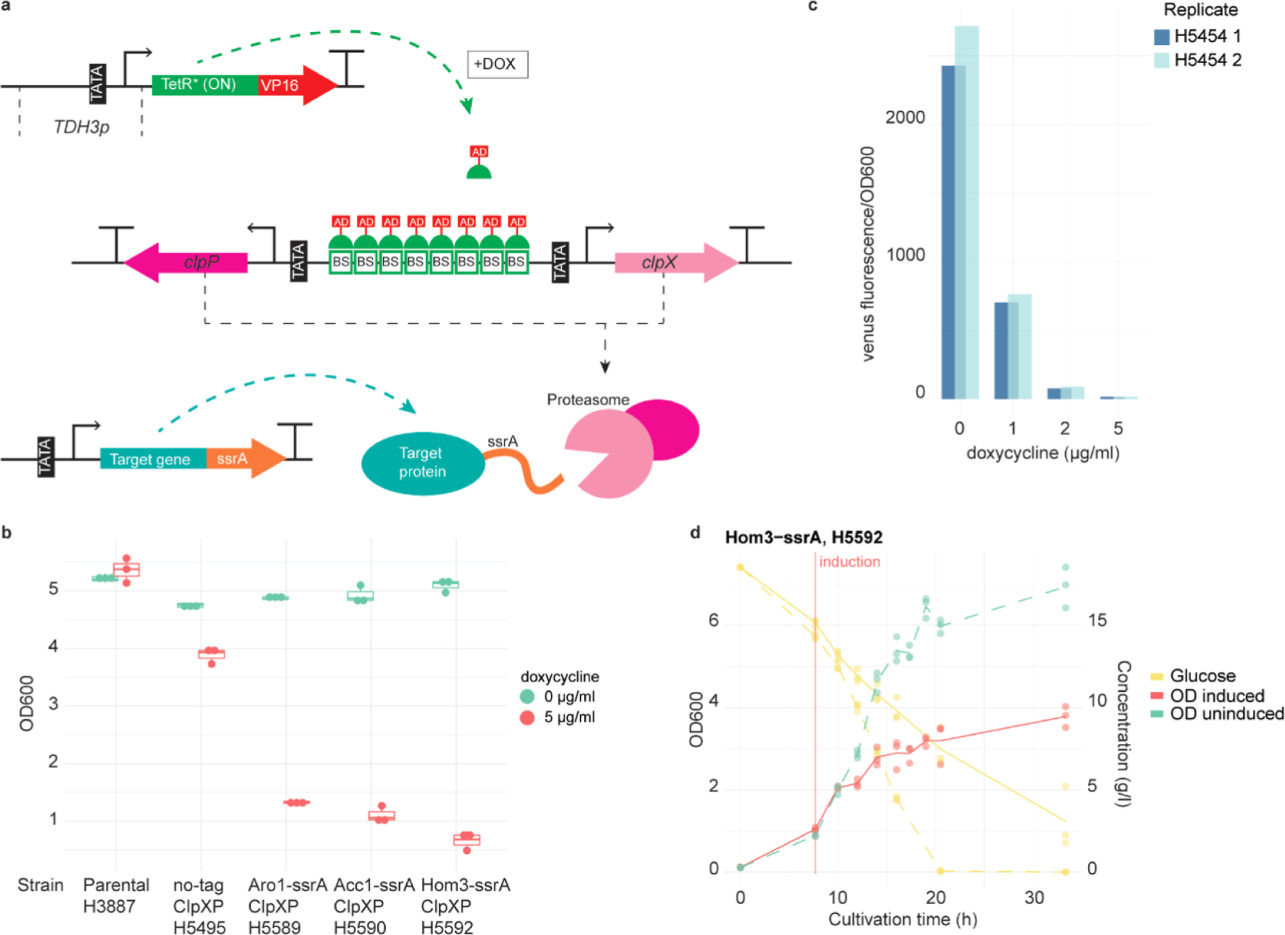
Inducible synthetic regulation system introduced into *S. cerevisiae*. (a) The core of the inducible synthetic regulation system is the ClpXP proteasome introduced under the Tet-On-inducible regulation. Tet-On is constitutively expressed from a strong *TDH*3 promoter. In the presence of doxycycline (DOX), the TetR-VP16 fusion protein binds the Tet binding sites (BSs)^37^ within the synthetic bidirectional promoter.^36^ Once bound, the VP16 activation domain recruits RNA polymerase, and transcription of the ClpP and ClpX components becomes active. The assembled ClpXP proteasome then recognizes proteins having an ssrA tag that was here introduced to the selected target proteins. (b) OD_600_ of three biological replicates per strain was reached with four different strains (H3887, H5495, H5590, and H5592), when 5 μg/mL DOX was added (red) or not (green) at inoculation. (c) Venus fluorescence in biological duplicate cultures of cells having the inducible ClpXP proteasome and ssrA tag introduced to the Venus protein when 0, 1, 2, or 5 μg/mL DOX was added. (d) Growth profiles and glucose consumption dynamics of three biological replicates of strain ssrA-tagged Hom3 ClpXP when the ClpXP proteasome was induced at 8 h with an addition of 5 μg/mL DOX (full lines; OD_600_ in red, glucose in yellow) or when it was not induced (dashed lines; OD_600_ in green, glucose in yellow).

We introduced the bacterial ClpXP proteasome that recognizes and degrades proteins tagged with the ssrA peptide (11 amino acid tag, AANDENYALAA)^33−35^ into *S. cerevisiae*. The ClpXP proteasome is composed of ClpX and ClpP proteins that were expressed from the Tet-On-dependent bidirectional promoter containing two core promoters^36^ spaced with eight TetR binding sites (Figure 1a). The low-mid strength core promoters^36^ were found to be ideal for use in the bidirectional promoter as they provided sufficiently strong protein degradation response, while the expression levels were low enough to limit substantial growth defects of the host (Figure 1b). The TetR-VP16 hybrid transactivator^37^ was expressed from a strong and constitutive *TDH*3 promoter (Figure 1a). To remove leakage of the original Tet-On system when uninduced, the previously identified point mutation was introduced into the original Tet-On sequence^37^ for changing glycine (GGG) at residue 72 to a valine (GTG).^38^

The functionality of the assembled ClpXP and Tet-On system was first tested with a product that can be easily expressed and directly quantified. Thus, the short ssrA tag for ClpXP proteasome recognition was added to the C-terminus of the sequence encoding Venus yellow fluorescent protein.^36^ The Venus-ssrA gene was placed under the control of the strong *TDH*3 promoter. The fluorescence of the yeast cells was measured after growth in different concentrations of DOX (DOX) in the culture medium (Figure 1c). Based on the results, the Venus fluorescence reduced in a DOX concentration-dependent manner, and the fluorescence was almost completely absent when a 5 μg/mL DOX concentration was used. Considering the high expression level of the Venus gene, this result indicates a highly efficient protein degradation capacity of the ClpXP system in*S. cerevisiae*.

Next, we assessed the effect of DOX addition and ClpXP expression on the growth of the yeast cells. As shown in Figure 1b, the addition of 5 μg/mL DOX does not affect the growth of the parental yeast strain (H3887) lacking both Tet-On and ClpXP expression cassettes. A similar result was obtained for another strain lacking these cassettes (Supporting Information, Figure S1). When the ClpXP expression was induced (with doxycycline 5 μg/mL) in a strain having Tet-On and ClpXP genes integrated but no ssrA-tagged proteins (H5495), we observed a growth reduction less than one OD_600_ unit compared to that in the uninduced state (Figure 1b). Other studies utilizing a protein degradation system have observed a 2-3-fold reduction in the maximum growth rate during the exponential phase when the system was activated in comparison to strains lacking the protein degradation system.^8,29^ In our case, the minor growth defect may be due to burden of heterologous ClpXP protein expression or off-target effects of the ClpXP proteasome.

### Synthetic Growth Control Achieved When Targeting Essential Metabolic Enzymes to the ClpXP Proteasome

Next, we tested whether control of growth could be achieved with the ClpXP proteasome under Tet-On induction. To that end, we created three*S. cerevisiae*strains that had the ClpXP proteasome under Tet-On induction and one of the three metabolic enzymes, either acetyl-CoA carboxylase Acc1, pentafunctional enzyme involved in aromatic amino acid synthesis Aro1, or aspartate kinase Hom3, expressed as ssrA-tagged. These three metabolic enzymes were arbitrarily chosen among the ones that lack isoenzymes and are growth-essential in synthetic defined medium (SDM) with glucose and ammonium as sole carbon and nitrogen sources, respectively, as identified by genome-scale metabolic model simulations and by assessing the null-mutant phenotype annotations (for viability) in the*S. cerevisiae*Genome Database (https://www.yeastgenome.org/). The three strains, each having one of the three metabolic enzymes ssrA-tagged [Acc1-ssrA (H5590), Aro1-ssrA (H5589), and Hom3-ssrA (H5592)], reached equally high OD_600_ within 0.5 OD_600_ as the control strain with ClpXP but no ssrA tag (H5495) in 18 h when Tet-On was not induced with DOX (equivalence test, *n*_control_ = 3, *n*_case_ = 9, *P* value < 0.001) (Figure 1b). All the strains with ClpXP integrated also reached as high as OD_600_ in 18 h as the parental strain (H3887) within 0.5 OD_600_ (equivalence test, *n*_control_ = 3, *n*_case_ = 12, *P* value 0.0090) (Figure 1b). The growth profiles of the strains without DOX did not differ from the growth profile of the parental strain (Supporting Information, Figure S2). Thus, efficient wild type growth was preserved when the ClpXP system was not induced with DOX. When 5 μg/mL DOX was introduced into the culture medium at inoculation, we observed substantially reduced OD_600_ levels (below 1.5 OD_600_) after 18 h of incubation in all three strains (Figure 1b).

Having shown the efficiency of the ClpXP proteasome in repressing ssrA-tagged enzyme activities, we next assessed whether inducible growth repression could be achieved. After inoculation (OD 0.1), we let the cells grow without DOX until the early exponential phase (OD ∼ 1). Then, we introduced DOX (5 μg/mL) in the culture medium to induce the ClpXP proteasome. Independent of which one of the three target metabolic enzymes (Acc1, Aro1, and Hom3) was ssrA-tagged for ClpXP proteasome recognition, growth repression was achieved (e.g., Hom3-ssrA, Figure 1d). Glucose consumption continued at growth repression but at the rate reduced to 7–10% of the uninduced exponential phase-specific glucose uptake rate (e.g., Hom3-ssrA, Figure 1d). Later, after 18 h of incubation, the growth reaccelerated (Supporting Information, Figure S3), which was likely an effect of a limited DOX half-life^39^ and confirmed the cultivability of cells after several hours of growth repression.

### Essential Target Enzyme Degradation Is Specific in Extensive Proteome Response

**Figure 2:**
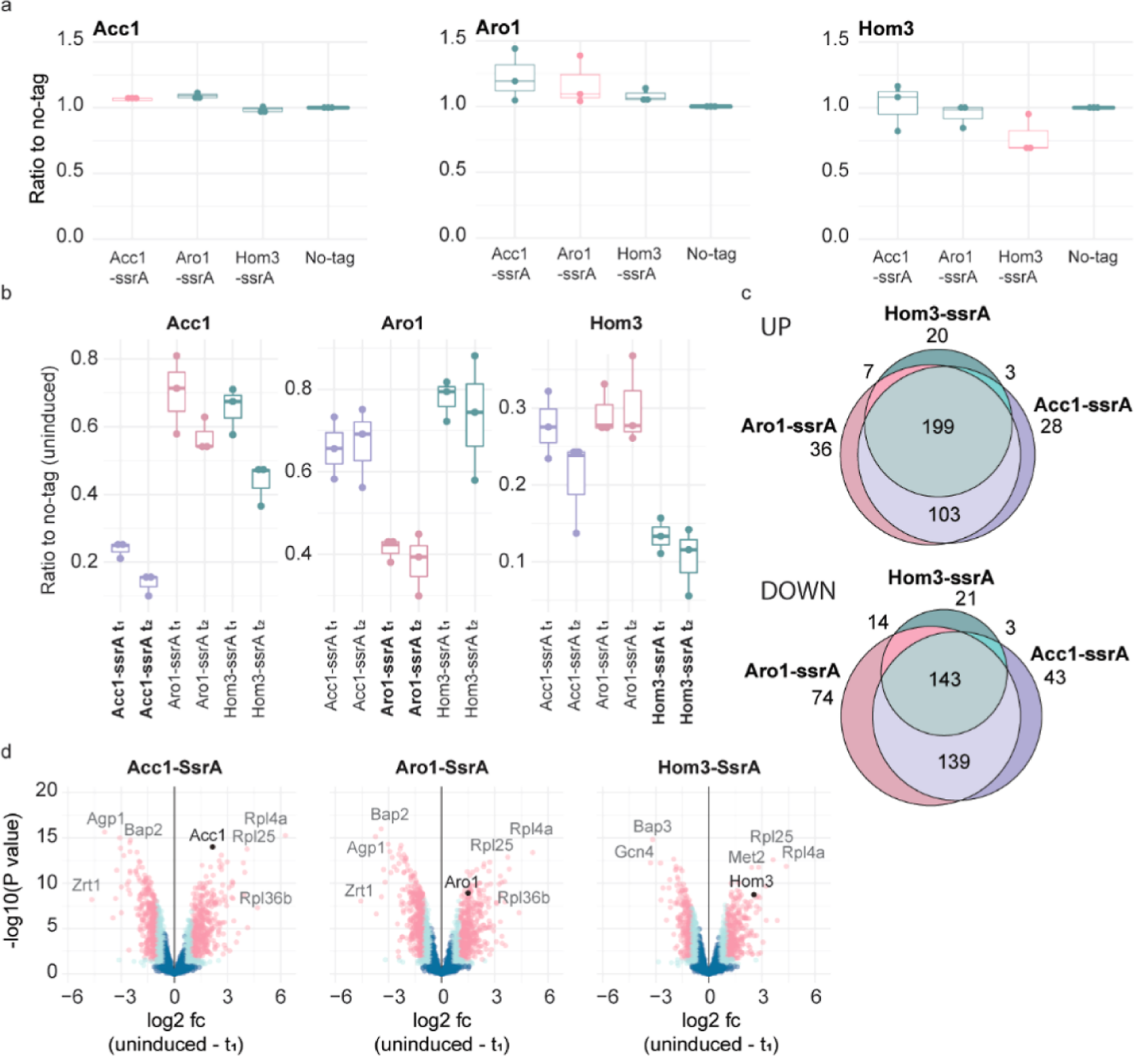
Relative quantification proteomics characterized*S. cerevisiae*proteome response to induced ClpXP target enzyme degradation and growth repression. (a) Relative abundances of three biological replicates of Acc1, Aro1, and Hom3 with respect to the particular protein abundances in the control strain (No-tag; no protein ssrA-tagged) in all the three strains in which one of the three enzymes (Acc1, Aro1, or Hom3) was ssrA-tagged and in the control strain (i.e., relative abundance one). The ssrA-tagged enzyme is highlighted in red. (b) Abundances of three biological replicates of Acc1, Aro1, and Hom3 relative to the control (No-tag, uninduced) in all three strains in which one of the enzymes (Acc1, Aro1, or Hom3) was ssrA-tagged at t_1_ (4 h after ClpXP induction) and t_2_ (23 h after inoculation). The sample identifier is highlighted in bold when the sample is from a strain in which the particular enzyme was ssrA-tagged. (c) Euler plots of sets of proteins found significantly higher (UP) and lower (DOWN) in abundance 4 h after induction of ClpXP proteasome expression compared to the uninduced state in three biological replicates when either Acc1, Aro1, or Hom3 was ssrA-tagged (limma; *n* = 3, fdr < 0.01, −1 > log2 fc > 1). (d) Volcano plots of relative quantification proteomics in three biological replicates of strains ssrA-tagged in either Acc1, Aro1, or Hom3, 4 h after inducing the ClpXP proteasome with respect to the uninduced state. Proteins with fdr < 0.05 and −2 > log2 fc > 2 (limma; *n* = 3) are shown in light red, the remaining proteins with fdr < 0.2 and −1.5 > log2 fc > 1.5 (limma; *n* = 3) are shown in light blue, and the rest of the quantified proteins are shown in dark blue.

Next, we assessed whether the target enzyme degradation by the ClpXP proteasome was specific when induced and how did the proteome as a whole respond. We performed relative quantification proteomics for characterizing the proteome status at ClpXP induction (i.e., uninduced samples), 4 h after induction, and 23 h after inoculation in the three strains expressing an ssrA-tagged essential metabolic enzyme. A control strain expressing ClpXP under Tet-On-dependent induction but having no proteins ssrA-tagged was cultured similarly, and a control sample was harvested at induction. Independent of which protein was ssrA-tagged, in uninduced states, the target protein levels were very similar to those of the control strain without an ssrA tag (Figure 2a). Thus, ClpXP activity did not leak when uninduced. In uninduced states, the total proteomes in the ssrA-tagged strains did not notably differ from those in the control strain without any protein being ssrA-tagged (i.e., only 3–4 proteins were differentially abundant, limma; *n* = 3, fdr < 0.01, −1 > log2 fc > 1, Supporting Information, Table S1). When the ClpXP proteasome was induced, the ssrA-tagged target protein levels (relative to the control) were specifically decreased both at 4 h after induction and 23 h after inoculation (Figure 2b). A major total proteome level response to ClpXP induction was observed as a high number of differentially expressed proteins independent of which essential metabolic enzyme was ssrA-tagged (Figure 2c,d). The responses were similar between the strains in terms of the differentially abundant proteins (Figure 2c) and the extent of the proteome responses (Figure 2d) indicating general regulatory events triggered in the cells, though in each strain, the enzyme targeted for degradation by the ClpXP proteasome was specifically repressed in abundance (Figure 2d). 4 h after induction, only a small number of proteins were differentially abundant between the strains (Acc1-ssrA vs Hom3-ssrA: 27, Aro1-ssrA vs Hom3-ssrA: 13, Aro1-ssrA vs Acc1-ssrA: 9, limma; *n* = 3, fdr < 0.01, −1 > log2 fc > 1). Further changes in the protein abundances during the induced states between 4 h after induction and 23 h after inoculation were notably smaller (Supporting Information, Figure S4).

### Heterologous cis,cis-Muconic Acid and Glycolic Acid Yield Improvement Is Achieved with Synthetic Growth Control

**Figure 3:**
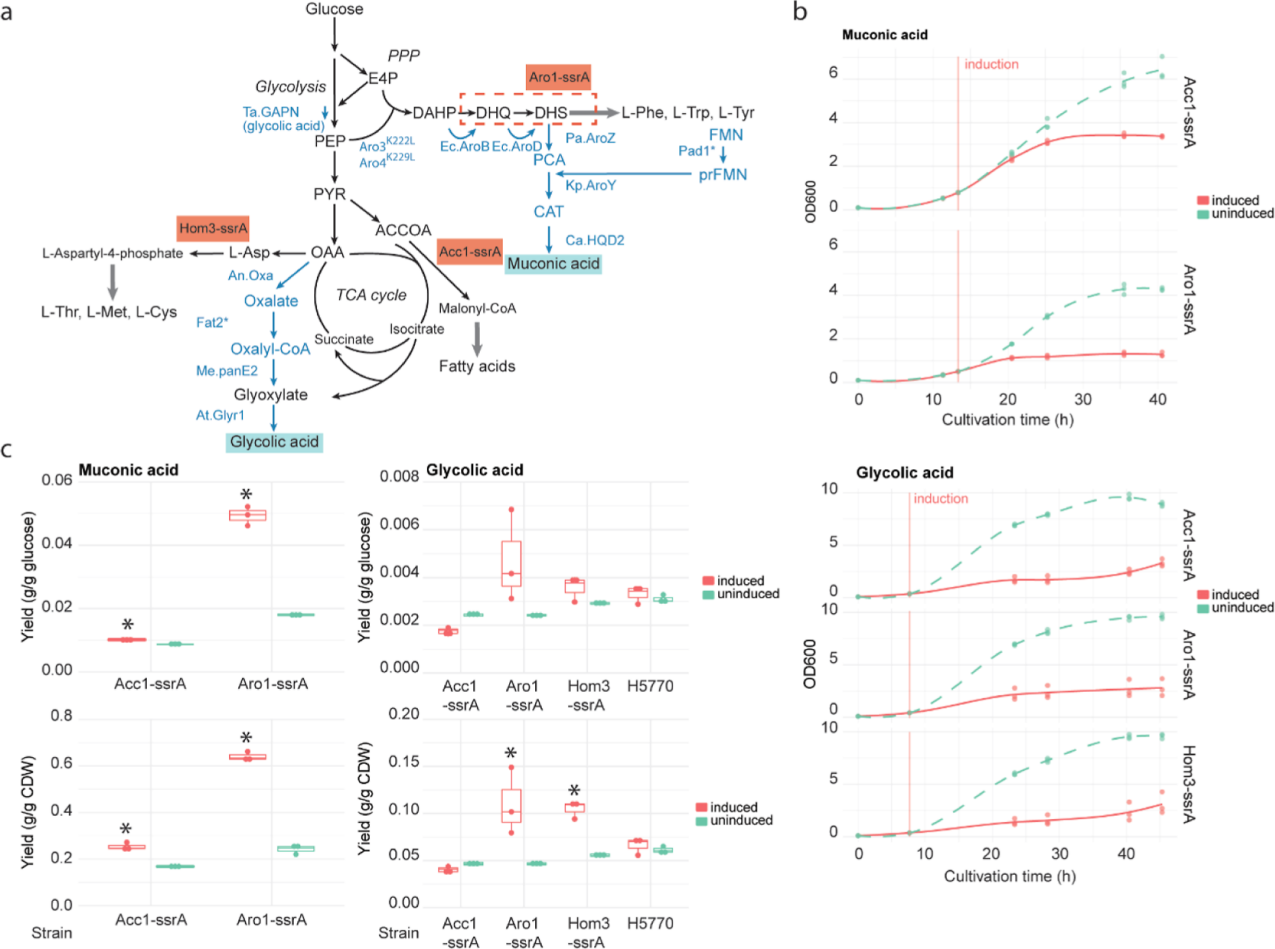
Inducible repression of growth-essential metabolic enzymes in MA- and GA-producing strains of *S. cerevisiae*. (a) Heterologous MA and GA synthesis pathways (blue arrows) utilize precursors from different locations of central metabolism. Heterologous genes and compounds involved are shown in blue. Essential (in SDM) metabolic enzymes Acc1, Aro1, and Hom3 targeted in individual strains for inducible degradation by the ClpXP proteasome. Aro1 activities for aromatic amino acid synthesis compete directly for precursors with the MA synthesis pathway. Hom3 (aspartate kinase) competes directly for precursors with the GA synthesis pathway. Acc1 (acetyl-CoA carboxylase) competes only indirectly for carbon resources with the two heterologous production pathways. (b) Growth profiles of strains having either the MA or GA production pathway and an essential metabolic enzyme ssrA-tagged when the ClpXP proteasome is either induced during exponential growth [at 13 h (MA) and 8 h (GA)] or not induced. The growth of cultures in which the ClpXP proteasome was induced and was not induced is shown in red and green, respectively. The growth curves are shown as *loess* fits to averages of three biological replicates (ClpXP induced: full line, ClpXP not induced: dashed line). (c) Effects of inducing inducible growth repression at 13 h (MA) and 8 h (GA) on the MA and GA yields from glucose and per biomass at 36 h (MA) and 40 h (GA). Yields of biological triplicate cultures in which the ClpXP proteasome was and was not induced are shown in red and green, respectively. The significantly improved yields are marked with stars (two-sided *t*-test; *n* = 3; g MA/g glucose: Aro1-ssrA *P* value < 0.001, Acc1-ssrA *P* value 0.017; g MA/g CDW: Aro1-ssrA *P* value < 0.001, Acc1-ssrA *P* value < 0.001; g GA/g CDW: Aro1-ssrA *P* value 0.010, Hom3-ssrA *P* value < 0.001).

We hypothesized that the effect of a single enzyme degradation-mediated growth repression on production may depend on how the growth-essential metabolic enzyme is localized in the metabolic network with respect to the production pathway. Direct effects would arise when a growth-essential enzyme that competes for a precursor(s) with the production pathway is degraded. On the other hand, growth repression by any essential enzyme degradation may indirectly release a higher proportion of cellular resources for product synthesis. Using genome-scale metabolic model simulations, we determined how the three metabolic enzymes (Acc1, Aro1, Hom3) are positioned, among other growth-essential metabolic enzymes, with respect to heterologous MA and GA pathways in yeast.^40,41^ The pathway for MA synthesis used was as in Pyne et al. (2018)^41^ and Brückner et al. (2018)^42^ with *Podospora anserina* 3-dehydroshikimate (DHS) dehydratase (Pa.AroZ), *Klebsiella pneumonia* protocatechuate (PCA) decarboxylase (Kp.AroY), *Candida albicans* catechol 1,2-dioxygenase (Ca.Hqd2), and FMN prenyltransferase (Pad1) from the *S. cerevisiae* reference strain S288C (Figure 3). The route was also complemented as per Brückner et al. (2018)^42^ with feedback regulation mutants of Aro3p and Aro4p and as per Pyne et al. (2018)^41^*Escherichia coli* Ec.AroB encoding 3-dehydroquinate (DHQ) synthetase and Ec.AroD encoding DHQ dehydratase, activities of the pentafunctional Aro1p. For GA synthesis, three heterologous activities [oxaloacetase (OXA) from*Aspergillus niger*, oxalyl-CoA reductase (panE2) from *Methylobacterium extorquens*, and glyoxylate reductase (GLYR1) from*Arabidopsis thaliana]* were introduced as proposed by Toivari et al. (2019).^40^ The route was complemented with the oxalate-CoA ligase (FAT2) from the*S. cerevisiae* reference strain S288c. In addition, nicotinamide adenine dinucleotide phosphate (NADP)-dependent glyceraldehyde-3-phosphate dehydrogenase (GapN) from*Triticum aestivum*was introduced to enhance NADPH availability for glyoxylate reductase. As an auxiliary enzyme, GapN was not considered part of the heterologous pathway for GA synthesis.

We calculated the distances of growth-essential enzymes not needed for production (see Materials and Methods for details) to the reactions of the heterologous MA and GA synthesis pathways as Simeonidis et al. (2003).^43^ Aro1 competes directly for the 3-hydroshikimate precursor with the MA synthesis pathway (i.e., distance of one to one of Aro1-catalyzed reactions) (Figure 3a) and is the only enzyme localized on this short distance. The GA synthesis pathway^40^ competes for the precursor oxaloacetate with Hom3 on two-reaction distance, and Aro1 is also localized on two-reaction distance to the GA synthesis pathway (Figure 3a, Supporting Information, Table S2). Acc1 is slightly more distant from the GA and MA pathways, 3 and 7 reactions, respectively. Hom3 distance to the MA pathway was 6 reactions.

To assess the effects of both direct and more distant growth-essential enzyme degradation on production, we created five strains by introducing the two heterologous pathways, for MA^41,42^ and GA^40^ synthesis, to the strains with the inducible ClpXP proteasome. The MA pathway was introduced into strains with ClpXP targeted to Aro1 (short distance) and Acc1 (long distance), and the GA pathway was introduced into strains with ClpXP targeted to Aro1, Acc1, and Hom3, all on short distance. Efficient growth repression was maintained in the strains with the MA and GA synthesis pathways with the essential metabolic enzyme ssrA-tagged (Figure 3b). Compared to that in the uninduced cultures, growth ceased independent of which of the essential metabolic enzymes was ssrA-tagged. The delay from the ClpXP induction by DOX addition to growth repression was dependent on the ssrA-tagged target metabolic enzyme. Growth repression was observed with shorter delay for the cells in which Aro1p or Hom3p was ssrA-tagged than that in cells with Acc1 ssrA-tagged. However, likely due to stronger reduction in the specific growth rate, the delays from ClpXP induction to growth repression were longer in the strains with the MA pathway than those in the strains with the GA synthesis pathway or without any heterologous product pathway (Figure 3b).

During growth repression, glucose consumption continued in all cultures, albeit at slower specific rates (Supporting Information, Figure S5). Ethanol accumulation was accordingly reduced. Simultaneously, the resource use for GA or MA synthesis appeared affected (Supporting Information Figures S6 and S7). The total GA yield on biomass by 40 h was found increased in ClpXP-induced cultures of strains with either Aro1 or Hom3 ssrA-tagged, whereas no yield increase was observed in induced cultures of strains with Acc1 ssrA-tagged, in comparison to uninduced strains (Figure 3c; at different time points: Supporting Information Figure S8). At late time points of the induced GA-producing cultures, cell growth was reinitiated (Figure 3b) as expected due to the DOX half-life (20 h). In the induced MA-producing cultures, growth reinitiation was not detected by 40 h, but similarly to the induced GA-producing cultures, likely growth preceding reacceleration of glucose consumption was observed after 36 h in the induced cultures of Aro1-ssrA (Supporting Information Figure S5). Nevertheless, in all cultures, GA and MA titers monotonically increased while the cells consumed glucose (Supporting Information Figures S6 and S7). By 36 h, the total MA yields on glucose and on biomass were found significantly increased when the ClpXP proteasome was induced to degrade either Aro1 or Acc1 with respect to the cultures in which it was not induced (Figure 3c; at different time points: Supporting Information Figure S8). In the cultures of the Aro1 ssrA-tagged strain, the yield increase was notably higher than that in the cultures of the strain having the ssrA tag in Acc1, which only indirectly competes for resources with the production pathway. Thus, MA and GA yields were particularly enhanced when inducing the degradation of the nearby growth-essential enzyme followed by growth repression. MA production has also previously been improved by deleting^44^ or inactivating^42^ Aro1 in the production strains. However, the inactivation results in auxotrophy, not ideal for industrial fermentations. To avoid auxotrophy, Pyne et al. (2018)^41^ reduced Aro1 activity by targeting it for degradation by the host proteasome. Although this improves yield, the resulting growth defect when amino acids were not available (i.e., lower growth rate and extended lag) is expected to substantially prolong batch times of production.

### Inducible Synthetic Protein Degradation System Efficient for Time-Separating Cell Growth and Production

Our results show that the synthetic regulation system with the inducible ClpXP proteasome is efficient for protein-level control of growth during cultivation processes in*S. cerevisiae*. Cessation of wild-type growth was achieved by inducing the ClpXP expression when any of the three metabolic enzymes (Acc1, Aro1, and Hom3), growth-essential in SDM, was targeted for degradation. The targeting in itself is unambiguous. The ssrA tag that the ClpXP proteasome recognizes does not require optimization in sequence. This is in contrast to gRNA optimization for CRISPRi gene expression repression which cannot yet be reliably predicted.^45^ To achieve the repression, the heterologous ClpXP proteasome contains a protein degradation module in contrast to the auxin-dependent protein degradation system^29^ that relies on the native proteasome. Thus, the ClpXP-dependent synthetic regulation leaves the native proteasome unoccupied to respond to the native cellular regulatory cues.

When main nutrients are depleted, the native regulation of*S. cerevisiae* halts resource utilization and ultimately takes the cells into quiescence.^21,22^ Thus, operationally simple nutrient-limited conditions cannot effectively be used for dynamic control over resource distribution between growth and production. In severe ammonium limitation, steady near-zero growth of*S. cerevisiae* (μ < 0.002 h^–1^, strain CEN.PK 113-7D) has been established,^20^ but simultaneously, the specific glucose utilization rate decreased to the bare minimum of ∼3% of the rate in an unlimited exponential growth (μ = 0.37 h^–1^, strain CEN.PK 113-7D).^46^ However, the low glucose utilization became decoupled from the near-zero growth delivering a product yield improvement.^47^ Growth of*S. cerevisiae*cells ceases also when supplemented auxotrophic nutrients are depleted, but this has been reported to trigger a phenotypic state rather than resembling stationary phase cells that can consume available glucose in contrast to quiescent cells.^48,49^ The corresponding glucose utilization rate may depend on the specific regulatory response that the conditional auxotrophy triggers.^50^ Immobilized*S. cerevisiae* cells unable to proliferate have also been found to maintain metabolic activity and be capable of fermenting available glucose even for a couple of weeks.^51^ Similarly,*S. cerevisiae*’s metabolic activity was found preserved here, i.e., specific glucose utilization at ∼10%, when growth was synthetically ceased by targeting the growth-essential metabolic enzymes to the ClpXP proteasome.

The growth repression enhanced the distribution of resources toward production of both MA and GA, as seen in increased yields, when the ClpXP-targeted enzyme was close in the metabolism to the heterologous pathways. To the best of our knowledge, the achieved MA yield from glucose is the highest reported in SDM (i.e., glucose and ammonium as carbon and nitrogen sources, respectively) in shake flasks. The previously achieved highest yield under similar conditions (without amino acid supplementation) was 8 mg/g.^44^ In fed-batch cultures of*S. cerevisiae*on complete or complex media, double as high heterologous MA yields^52^ and substantially, 10–110-fold higher titers, as usual for fed-batch cultures, have been achieved^52−54^ than here on SDM in shake flasks. Other hosts may also be prominent MA producers as notable titers of up to 3^55^ or 4^56^ times higher have been reached than the maximum titers achieved with *S. cerevisiae*. With*E. coli*on synthetic defined media without amino acids (xylose or glucose as the carbon source), GA titers of 40 g/L^57^ or 60 g/L^58^ have been reached. However, GA titers or yields (without amino acid supplementation) on glucose in*S. cerevisiae*have not been reported before,^59,60^ though a direct conversion of glucose is desirable to avoid the carbon loss in fermentation. The highest previous titer of GA (∼20 mg/L) involving glucose utilization had been obtained when glucose was used together with xylose as carbon sources in synthetic complete medium.^61^ This titer increased then after the ethanol utilization phase started and reached the highest level of 0.15 g/L at 168 h of cultivation. Comparable titers (0.03–0.12 g/L) have also been reported by Koivistoinen et al. (2013).^60^ Here, a similar titer of 0.15 g/L was achieved directly from glucose in ∼40 h by using the ClpXP growth regulation.

The synthetic growth regulation using ClpXP can be extended to other products by selecting suitable degradation targets. We showed here how this can be done using genome-scale metabolic model simulations, which are generalizable across hosts and products. Genome-scale metabolic model simulations have been successfully used for predicting metabolic gene/reaction essentiality and designing strains for overproduction.^62,63^ In addition to predicting essentiality for growth and production, we considered the distance of degradation target enzymes from the production pathways. Our demonstration cases suggest that short pathway distance between the production pathway and degradation target could be beneficial for product yield improvement.

## Conclusions

Improving production to an economically attractive level is a major challenge in developing novel industrial processes that use microbial cells for chemical synthesis. Inducible growth regulation and two-stage processes for time-separated growth and production phases have been proposed as a solution for improving productivity in particular.^17^ However, the specific substrate utilization rate commonly decreases with the growth rate, as we observed here, and may diminish the benefit of the time separation of growth and production.^64^ While the challenge of decreased substrate utilization remains, our results show that the inducible repression of a growth-essential metabolic enzyme alone enables us to improve product yields. We further noted that the initial hours after triggering the synthetic regulation are the most beneficial for the production which is to be contemplated in the next developments. The inducible ClpXP proteasome has potential to be used for such synthetic regulation in*S. cerevisiae,* encouraging the development of and coupling with induction methods compatible with large-scale processes.

## Materials and Methods

### Genome-Scale Metabolic Model Simulations

Genome-scale metabolic model simulations were performed with *S. cerevisiae* consensus model v. 7.6^65^ (currently hosted at https://github.com/SysBioChalmers/yeast-GEM) and Matlab v. 9.3.0 (R2017b) with IBM cplex v. 12.8.0 (https://www.ibm.com/products/ilog-cplex-optimization-studio) as the linear programing/mixed-integer linear programing solver. Cobra toolbox v. 2.13.3 was used for the model manipulations, while the simulations were performed using in-house-implemented programs. Flux balance analysis (FBA)^66^ was used for identifying growth-essential metabolic reactions in*S. cerevisiae*consensus model v. 7.6 under SDM conditions with glucose and ammonium as sole carbon and nitrogen sources, respectively. Single-enzyme-catalyzed growth-essential reactions were further selected based on the model’s gene annotations. Among these enzymes, acetyl-CoA carboxylase encoded by Acc1, pentafunctional enzyme catalyzing multiple steps of chorismate biosynthesis encoded by Aro1, and aspartate kinase encoded by Hom3 were selected as inducible degradation target enzymes for this project.

Classification of the enzymes targeted to ClpXP with respect to the MA and GA pathways was performed using simulations of the genome-scale metabolic model of *S. cerevisiae* v. 8.6.0 (https://sysbiochalmers.github.io/yeast-GEM/) and pathway distance calculation.^43^ The pathway distance calculation was implemented using Python v. 3.8 with the IBM ILOG CPLEX v. 12.10.0 solver and *docplex* package v. 2.23.222 (https://github.com/ptjouhten/ClpXP). Highly connected metabolites were removed from the metabolic network before the distance calculations (Table S8). The heterologous pathways were introduced to the model using *cobra* v. 0.23.0, and *reframed* v. 1.2.1 was used for running parsimonious FBA (pFBA) for identifying reactions needed for optimal production and linear minimization of metabolic adjustment simulations for identifying the reaction that is instead essential for growth (at least 90% of optimum).

### Medium and Cultivation Conditions

*E. coli* was grown in lysogeny broth (LB) with 100 μg/mL ampicillin, 50 μg/mL kanamycin, or 25 μg/mL chloramphenicol (MERCK). For selection and maintenance of plasmids, selective yeast extract peptone dextrose medium (YPD) containing 20 g/L peptone (VWR Chemicals), 10 g/L yeast extract (OXOID), 20 g/L d-glucose (VWR Chemicals), and 200 μg/mL nourseothricin dihydrogen sulfate (NAT) (Jena Bioscience, AB-101) and/or 200 μg/mL Geneticin G-418 sulfate powder (G418) (MERCK) was used.

For fluorescence measurement, strain H5454 was cultured in duplicate synthetic complete dextrose medium without leucine and uracil (SCD-LEU-URA). Solution contained 6.7 g/L yeast nitrogen base w/o amino acids (YNB, BD Diagnostic Systems), 20 g/L d-glucose (VWR Chemicals), and 50 mL/L amino acid stock solution (Table 1).

**Table 1:** Content of Amino Acid Stock Solution Used for SC-Minus Medium

amino acid	g/L
adenine	0.0405
arginine	1.044
aspartic acid	0.798
histidine	0.174
myo-inositol	0.108
isoleucine	1.575
leucine	0.786
lysine	0.273
methionine	0.447
Phenylalanine	0.249
serine	0.315
threonine	0.357
tryptophan	0.246
tyrosine	0.09
uracil	0.066
valine	0.351

For other experiments, *S. cerevisiae* strains were grown in biological duplicate or triplicate at 30 °C with 200–220 rpm shaking in SDM: 6.7 g/L YNB w/o amino acids (BD Diagnostic Systems) and 20 or 40 g/L d-glucose (VWR Chemicals). For the GA strains, 0.5x pH 5.6 synthetic defined medium with ammonium sulfate (SD-AS) w/o amino acids with 40 g/L d-glucose was used.^67^ Cultures were induced with 1, 2, 5, 10, or 20 μg/mL doxycycline (MERCK).

### Plasmid Construction

The plasmids and oligonucleotides employed in this study are listed in Tables S3–S5, with the B-number referring to VTT’s internal collection. Restriction enzymes were obtained from Thermo Scientific (USA), New England BioLabs (USA), and Roche (Switzerland). Oligos were ordered from MERCK Life Science Oy (Germany) or Integrated DNA technologies (IDT) (USA). Amplified polymerase chain reaction (PCR) products and digested DNA fragments were purified before cloning with gel electrophoresis using 0.8% agarose and a QIAquick PCR Purification Kit (Qiagen, Netherlands) or with a Monarch PCR & DNA Cleanup Kit (New England BioLabs). Plasmid isolation was done with the Thermo Scientific DNA extraction kit.

The single-guide RNA plasmids for introduction of the ssrA tag into the target proteins were constructed by introducing crRNA fragments into a gRNA expression plasmid (B9544). This plasmid contained sequences required for propagation and selection in *E. coli* (*ampR*) and *S. cerevisiae* (*NAT*) and a pSNR52 promoter and tracrRNA spaced with a *MssI* restriction site enabling linearization of the plasmid. The introduced crRNA fragments contained homologous flanks to the pSNR52 promoter and the tracrRNA in addition to the actual crRNA sequence.

The crRNA fragments were assembled by annealing two single-stranded oligos in duplex buffer (IDT). First, the oligos were incubated for 5 min at +95 °C, and then, the temperature was decreased by 0.1 °C/s for 20 min. The purpose of using single-stranded oligos for fragment assembly was to demonstrate the possibility of constructing large gRNA libraries cost-efficiently (corresponding readily bought double-stranded DNA fragments would have been significantly more expensive). The resulting double-stranded crRNA fragments were transformed into yeast H3887 together with the linearized gRNA expression plasmid to generate a circular plasmid utilizing yeast homology recombination. Once assembled in yeast, the crRNA and tracrRNA created a functional single-guide RNA controlled by a pSNR52 promoter. The assembled gRNA plasmids were isolated from yeast using the phenol–chloroform extraction method, and the plasmids were transformed into *E. coli* for plasmid propagation.

The donor DNA fragments (see chapter Strain Construction) used together with the corresponding gRNA fragments were prepared in a similar way to the crRNA fragments by annealing single-stranded DNA oligos (120 bp each). However, in the case of donor DNAs, the oligos were only partially complementary because the donor DNA fragments were longer than the crRNA fragments. To extend the fragments to being fully double-stranded, Kapa HiFi enzyme ready mix (Kapa Biosystems) was applied after annealing the oligos. As in the case of crRNA fragments, the purpose of using oligos for generation of double-stranded DNA fragments was to demonstrate that short double-stranded DNA fragments can be prepared economically. This can be advantageous if large donor DNA libraries need to be prepared.

The plasmids required for the MA pathway were constructed with the Golden Gate technology-based modular cloning (MoClo) system using Type IIS BsmBI (R0580L, New England BioLabs) and BsaI (R0535L, New England BioLabs) restriction enzymes,^68^ Phusion U Hot Start DNA Polymerase (F555S, Thermo Scientific), and uracil-specific excision reaction-based cloning technique (USER enzyme, New England BioLabs).^69^ The Tet-On, ClpXP, GA, and other plasmids were constructed according to the manufacturer’s protocol using Gibson Assembly (E2611S, New England BioLabs) or restriction enzyme-based techniques (Thermo Fisher Scientific). All ligation and Gibson Assembly mixes were transformed into*E. coli* TOP10 by electroporation.^70^ Plasmids were verified with analytical digestion and Sanger sequencing (Eurofins Scientific SE).

### Phenol–Chloroform DNA Extraction

The phenol–chloroform method was used to extract gRNA plasmids from yeast and to extract genomic DNA for copy number analysis. For extraction, 600 μL of glass beads, 600 μL of 1xTE (pH 7.5), and 600 μL of phenol–chloroform–isoamyl alcohol solution (50% phenol, 48% chloroform, and 2% isoamyl alcohol) were mixed with cells. A Precellys 24 homogenizer (Bertin Instruments) was used for two rounds of bead beating: 30 s at 6.5 mZ. For copy number analysis, the aqueous layer was 100x diluted in distilled de-ionized water (DDIW) and used as a template for the quantitative real-time PCR (qPCR) reaction.

### Strain Construction

The yeast strains used and constructed in this work are listed in Table S6, with the H-number referring to VTT’s internal collection.*S. cerevisiae* CEN.PK parent strains were kindly provided by Dr. Kötter (Institut für Mikrobiologie, J.W. Goethe Universität, Frankfurt, Germany). The heterologous genes integrated into strains are listed in Table S7.

All*S. cerevisiae*transformations were done using the standard lithium acetate protocol,^71^ with expression plasmids linearized by NotI enzyme (FD0596, Thermo Scientific). The EasyClone expression plasmids were transformed into yeast cells using the CRISPR/Cas9 protocol of the EasyClone kit^72^ and lithium acetate method. Correct integration was confirmed with DreamTaq PCR (Thermo Scientific) and Sanger sequencing.

CRISPR/Cas9 was used to introduce the ssrA tag (11 amino acid tag) to the target protein’s C-terminus. Thus, the gRNA was designed to introduce a double-stranded break in the 3′ end of the target gene, close to a stop codon (within the ORF). The gRNAs were expressed from a plasmid with the NAT selection marker gene (B9974, B9997, and B9999), and the Cas9 protein was expressed from the plasmid B7770, containing the G418 resistance gene.^73^

The transformed donor DNA included an integration flank homologous to upstream of the double-stranded break, the ssrA tag, a stop codon, and an integration flank homologous to downstream of the stop codon (terminator sequence), respectively. In addition, to restore the native amino acid sequence between the double-stranded break site and the stop codon, the missing sequence was introduced between the 5′ flank and the ssrA tag. The ssrA tag was placed in a frame with the gene and was immediately followed by a stop codon. To prevent repetitive digestion of the recombined donor DNA by the Cas9 nuclease, the protospacer adjacent motif site of the protospacer was removed in the designed donor DNA with an alternative codon.

To generate the strains with a target protein ssrA-tagged, the gRNA plasmids were co-transformed with a corresponding donor DNA fragment into Cas9-expressing yeast strain H5498. The correct introduction of the ssrA tag was confirmed by analytical PCR. The analytical PCR products with expected sizes were sent for DNA sequencing. To remove the gRNA and Cas9 expression plasmids from the final strains, they were repetitively plated without NAT and G418 selection. After a few rounds of plating, the absence of gRNA and Cas9 plasmids was confirmed by showing that the strains were unable to grow under NAT or G418 selective conditions.

The MA pathway was constructed as in Pyne et al. (2018)^41^ and Brückner et al. (2018).^42^ The following genes were codon-optimized for expression in*S. cerevisiae*, synthesized by IDT and cloned into EasyClone expression vectors: *Pa*.*AROZ*, *Ca*.*HQD*2, *Kp*.*AROY*, *PAD*1 from*S. cerevisiae*strain S288C, *Ec.AROB, Ec.AROD, ARO*3^*K*222*L*^, and *ARO*4^*K*229*L*^ from*S. cerevisiae*CEN.PK.

The GA pathway was constructed as in Toivari et al. (2019),^40^ with the *FAT*2, *OXA*, *panE*2, *GLYR*1, and *GAPN* Gibson Assembly cassettes cloned into EasyClone expression vectors.

### Copy Number Analysis

Copy number analysis of transformed genes was done using qPCR with inorganic pyrophosphatase (*IPP*1) as the reference gene. LightCycler 480 SYBR Green I Master (Roche) qPCR was used with LightCycler 480II (Advanced Relative Quantification Tool, Roche) according to manufacturer’s protocols. Analysis was done using accompanying software (Advanced Relative Quantification tool). The *S. cerevisiae* clones with correct genetic parts integrated (ClpP, ClpX, Tet-On, and ssrA tag) were chosen for MA and GA pathway transformations.

### HPLC Metabolite Analysis

For metabolite analysis, yeast cells were removed from the supernatant (1.2 mL) by centrifugation at room temperature (3 min, 4000 rpm). Concentrations of metabolites were measured by high-performance liquid chromatography (HPLC). The metabolites were separated by reversed-phase HPLC using the Alliance Water 2690 separation module. The column was eluted with 0.005 mol/L H_2_SO_4_ as the mobile phase and a flow rate of 0.5 mL/min. The detection of glucose, glycerol, ethanol, and acetate was done by means of a Shodex RI-101 refractive index detector. A UV detector (wavelength = 210 and 260 nm) was used for acetate and GA. For data evaluation, Empower software was used with two technical replicates per sample. All measurements were compared to linear standard curves of reference standards (MERCK). Standards and sample dilutions were prepared in DDIW.

### UPLC-MS Analysis of MA

The cell culture supernatant samples were analyzed for MA using a Waters Acquity UHPLC system (Milford, MA, USA) and Waters Xevo TQ-XS MS system (Milford, MA, USA), with two technical replicates per sample. Chromatography was performed using an ACQUITY UPLC BEH HSS T3 column, 1.8 μm, 2.1 mm × 100 mm (Waters) kept at 45 °C. The experiment was carried out at a flow rate of 0.4 mL/min with mobile phases A (0.1% formic acid in water) and B (0.1% formic acid in methanol). The gradient program was as follows: 0 min 5% B, 4.3 min 60.9% B, 5.6 min 97.1% B, and 6 min 95% B and equilibrium time between runs was 3.0 min. Injection volume was 2 μL. Mass spectrometry was carried out using electrospray ionization (ESI) in negative polarity. The capillary voltage was 2.1 kV, cone voltage 20 kV, source temperature 120 °C, and desolvation temperature 500 °C. The cone and desolvation gas flows were set at 150 L/h (nitrogen) and 1000 L/h (nitrogen), respectively. MA was detected using selected ion monitoring at *m*/*z* 141.

### GC-MS Analysis of GA

Cell culture supernatant samples (100 μL) were spiked with internal standard heptanoic acid and were evaporated to dryness under nitrogen flow. Thereafter, the samples were derivatized with 50 μL of *N*-methyl-*N*-(trimethylsilyl) trifluoroacetamide and 50 μL of pyridine at 60 °C for 60 min. The calibration curve was prepared for GA (Sigma-Aldrich), and the standards were prepared as the samples.

The samples were run on an Agilent 7890 gas chromatograph combined with an Agilent 5975 mass selective detector, with two technical replicates per sample. The injection volume was 1 μL. The inlet temperature was 250 °C, and the oven temperature program was from 60 to 290 °C. The analyses were performed on an Agilent DB-5MS capillary column (30 m, ID 250 μm, film thickness 0.25 μm).

### Fluorescent Strain Measurements

Cells were cultivated in duplicate on SCD-LEU-URA with 0, 1, 2, or 5 μg/mL doxycycline for 20 h (30 °C, 800 rpm). Cells were centrifuged, resuspended in 200 μL of DDIW, and transferred to Black Cliniplate (Thermo Fisher Scientific). Venus (yellow fluorescent protein) fluorescence was measured with Varioskan (Thermo Electron Corporation) using excitation and emission wavelengths of 510/530 nm (measurement time = 100 ms). A 100x dilution of the cell suspension was made for OD_600_ measurement with Varioskan (photometric measurement mode, wavelength = 600 nm, bandwidth = 5 nm, measurement time = 100 ms) using a transparent microtiter plate (Nunc 96F, Thermo Fisher Scientific), to normalize fluorescence measurement originating from different cell densities. The arbitrary units (AUs) reported in figures were obtained by dividing the fluorescence measurement value by the OD_600_ value.

To monitor the effect of DOX on growth, the same cells were used to inoculate 20 mL of SCD-URA-LEU medium, with 0, 1, 2, or 5 μg/mL DOX. Cells were incubated at 30 °C at 200 rpm for around 17 h with OD_600_ measurements taken regularly.

### Extraction of Proteins

The inoculum was prepared in SDM in biological triplicate. Each cell culture (3 mL) was transferred into ice-cold 15 mL falcon tubes and centrifuged (4 °C, 4000 rpm, 3 min). The cell pellets were washed with phosphate buffered saline buffer (3 mL) and frozen in liquid nitrogen, to be stored at −80 °C until extraction. Cell pellets were then resuspended in 1 mL of breaking buffer (10 mM Tris, 1 mM EDTA, 100 mM NaCl 2%, TritonX-100, 1% SDS) with addition of proteinase inhibitors aprotinin (MERCK) and leupeptin (MERCK) 1 μL each per 10 mL of breaking buffer. The extraction was performed on ice. The volumes were transferred to tubes with beads for three rounds of bead beating followed by the Precellys24 homogenizer (Bertin Instruments): 20 s at 5.5 mZ with 1 min cooling intervals on ice. To collect the supernatant, the suspensions were centrifuged for 3 min at full speed (15,000 rpm). Benzonase (25U, MERCK) was then added to the lysates, and they were incubated for 30 min at 37 °C.

### Proteomics Sample Preparation

Reduction of disulfide bridges in cysteine-containing proteins was performed with dithiothreitol (56 °C, 30 min, 10 mM in 50 mM HEPES, pH 8.5). Reduced cysteines were alkylated with 2-chloroacetamide (room temperature, in the dark, 30 min, 20 mM in 50 mM HEPES, pH 8.5). Samples were prepared using the SP3 protocol,^74,75^ and trypsin (sequencing grade, Promega) was added in an enzyme to protein ratio of 1:50 for overnight digestion at 37 °C.

Peptides were labeled with the TMT10plex^76^ isobaric label reagent (Thermo Fisher) according to the manufacturer’s instructions. Samples were combined for the TMT10plex, and for further sample cleanup, an OASIS HLB μElution plate (Waters) was used. Offline high-pH reverse-phase fractionation was carried out on an Agilent 1200 Infinity HPLC system, equipped with a Gemini C18 column (3 μm, 110 Å, 100 × 1.0 mm, Phenomenex).^77^

### Proteomics LC-MS/MS

After fragmentation, peptides were separated using the UltiMate 3000 RSLC nano LC system (Dionex) fitted with a trapping cartridge (μ-Precolumn C18 PepMap 100, 5 μm, 300 μm i.d. × 5 mm, 100 Å) and an analytical column (nanoEase M/Z HSS T3 column 75 μm × 250 mm C18, 1.8 μm, 100 Å, Waters). The outlet of the analytical column was coupled directly to a QExactive plus (Thermo) using the Proxeon nanoflow source in the positive ion mode. The peptides were introduced into the mass spectrometer (QExactive plus, Thermo Fisher) via a Pico-Tip Emitter 360 μm OD_600_ × 20 μm ID; 10 μm tip (New Objective), and a spray voltage of 2.3 kV was applied. The capillary temperature was set at 320 °C. Full scan mass spectrometry (MS) spectra with mass range 375–1200 *m*/*z* were acquired in the profile mode in the FT with a resolution of 70,000. The peptide match algorithm was set to “preferred” and charge exclusion “unassigned”, and charge states 1 and 5–8 were excluded. The isolation window was set to 1.0 and 100 *m*/*z* set as the fixed first mass. MS/MS data were acquired in the profile mode.^78^

Acquired data were processed using IsobarQuant^79^ and Mascot (v2.2.07) and searched against the UniProt *S. cerevisiae* CEN.PK113-7D proteome database. The following modifications were included into the search parameters: carbamidomethyl (C) and TMT10 (K) (fixed modification) and acetyl (N-term), oxidation (M), and TMT10 (N-term) (variable modifications). For the full scan (MS1), a mass error tolerance of 10 ppm was set, and for MS/MS (MS2) spectra, a mass error tolerance of 0.02 Da was set. Further parameters were set as follows: trypsin as the protease with an allowance of maximum two missed cleavages and a minimum peptide length of seven amino acids. At least two unique peptides were required for a protein identification. The false discovery rate on peptide and protein levels was set to 0.01.

Raw data of IsobarQuant were loaded into R. Only proteins that were quantified with two unique peptides were used for downstream analysis. The output data from IsobarQuant were cleaned for potential batch effects with limma^80^ and subsequently normalized with vsn (variance stabilization).^81^ Missing values were imputed with the impute function (method = “knn”) from the MSNBase package.^82^ Under these conditions, a total of 3305 proteins were quantified and used to calculate differential protein abundances between tested strains. Differential abundance was performed with limma.^80^ Proteins were classified as “hits” with a false discovery rate (fdr) of ≤ 5% and a fold change of at least 200% and as “candidates” with fdr ≤ 20% and a fold change of at least 100%.

### Data Analysis and Visualization

Data analysis and visualization were performed using R v. 4.1.2^83^ and packages *ggplot*2 v. 3.3.5,^84^*eulerr* v. 4.1.3,^85^ and *TOSTER* v. 0.6.0.^86^

## Supporting Information Available

Visualizations of strain growth, substrate utilization, production, proteome status, oligos, plasmids, strains, heterologous genes, and highly connected metabolites (PDF)
Proteomics differential relative abundance limma analysis results for all proteins appearing in at least one biological replicate (TXT)
Metabolic reaction distances to heterologous MA and GA synthesis pathways (XLSX)

## Notes

The MS proteomics data have been deposited to the ProteomeXchange Consortium via the PRIDE^87^ partner repository with the data set identifier PXD034451.— The modeling computer scripts are shared in GitHub (https://github.com/ptjouhten/ClpXP).
