# Supplementary material for: Inducible Synthetic Growth Regulation Using the ClpXP Proteasome Enhances cis,cis-Muconic Acid and Glycolic Acid Yields in Saccharomyces cerevisiae
Source: ACS Synth Biol. 2023 Mar 28;12(4):1021–33. doi: 10.1021/acssynbio.2c00467 (PMC10127448; doi:10.1021/acssynbio.2c00467)
Supplement: Supplementary file 1 — sb2c00467_si_001.pdf [file sb2c00467_si_001.pdf]

Supporting Information

Synthetic dynamic growth regulation using ClpXP proteasome enhances cis,cis-muconic acid and glycolic acid yields in *Saccharomyces cerevisiae*

Natalia Kakko[a,b,*], Anssi Rantasalo[a,c,*], Tino Koponen[a], Virve Vidgren[a], Matti Kannisto[a], Natalia Maiorova[a], Heli Nygren[a], Dominik Mojzita[a], Merja Penttilä[a,b], Paula Jouhten[a,b,!]

[a]VTT Technical Research Centre of Finland Ltd, 02044 VTT, Espoo, Finland

[b]Aalto University, School of Chemical Engineering, Department of Bioproducts and Biosystems, P.O. Box 16300, FI-00076 AALTO, Espoo, Finland

[c]Current address: EniferBio, anssi.rantasalo@eniferbio.fi

*These authors contributed equally

[!]Corresponding author

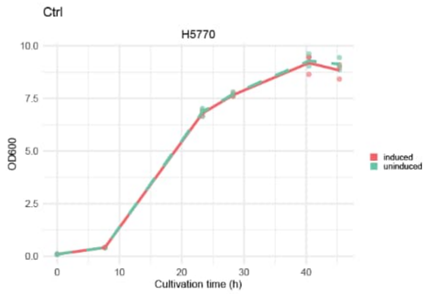

Figure S1. Growth profiles of a strain with a glycolic acid synthesis pathway but no ClpXP proteasome with and without doxycycline addition (H5770). Growth curves of cultures in which doxycycline was (at 8 h) and was not added are shown in red and green, respectively. The growth curves are shown as loess fits (uninduced dashed, induced full line) to averages of three biological replicates.

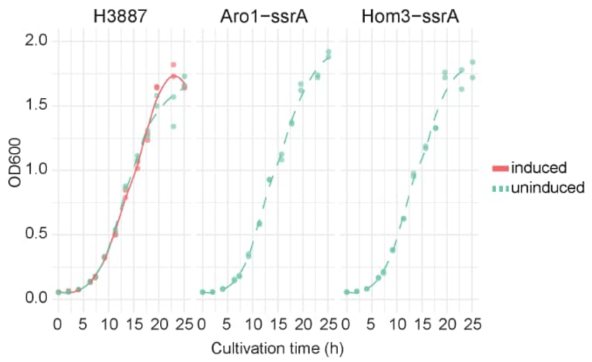

Figure S2. Growth profiles of a control strain (H3887) and strains with ClpXP proteasome and an essential metabolic enzyme ssrA-tagged (Aro1-ssrA, Hom3-ssrA). Growth profile of a culture with doxycycline added at inoculation is shown in red. The growth curves are shown as loess fits (uninduced dashed, induced full line) to two biological replicates.

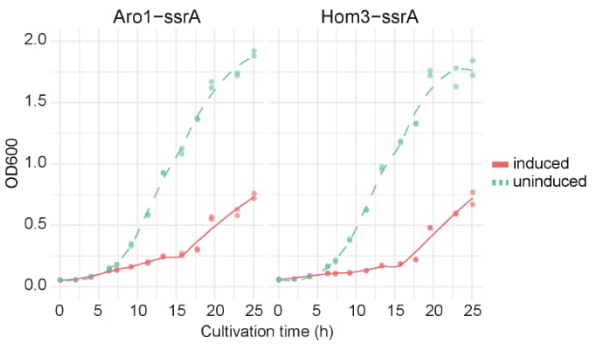

Figure S3. Growth profiles of strains with ClpXP proteasome and an essential metabolic enzyme ssrA-tagged when doxycycline for ClpXP induction was added at inoculation. Growth profiles of cultures in which the ClpXP proteasome was and was not induced are shown in red and green, respectively. The growth curves are shown as loess fits (uninduced dashed, induced full line) to two biological replicates.

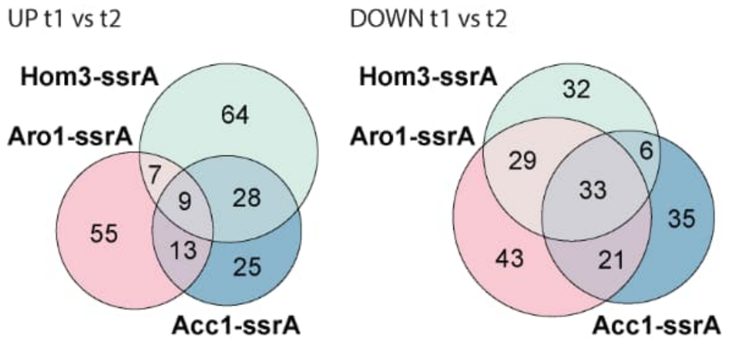

Figure S4. Significantly higher and lower abundant proteins (limma; n=3, fdr < 0.01, -1 > log2 fc > 1) between the initial response to ClpXP proteasome induction (4 h after) and after 23 h of incubation of three biological replicates when either Acc1, Aro1, or Hom3 was ssrA-tagged.

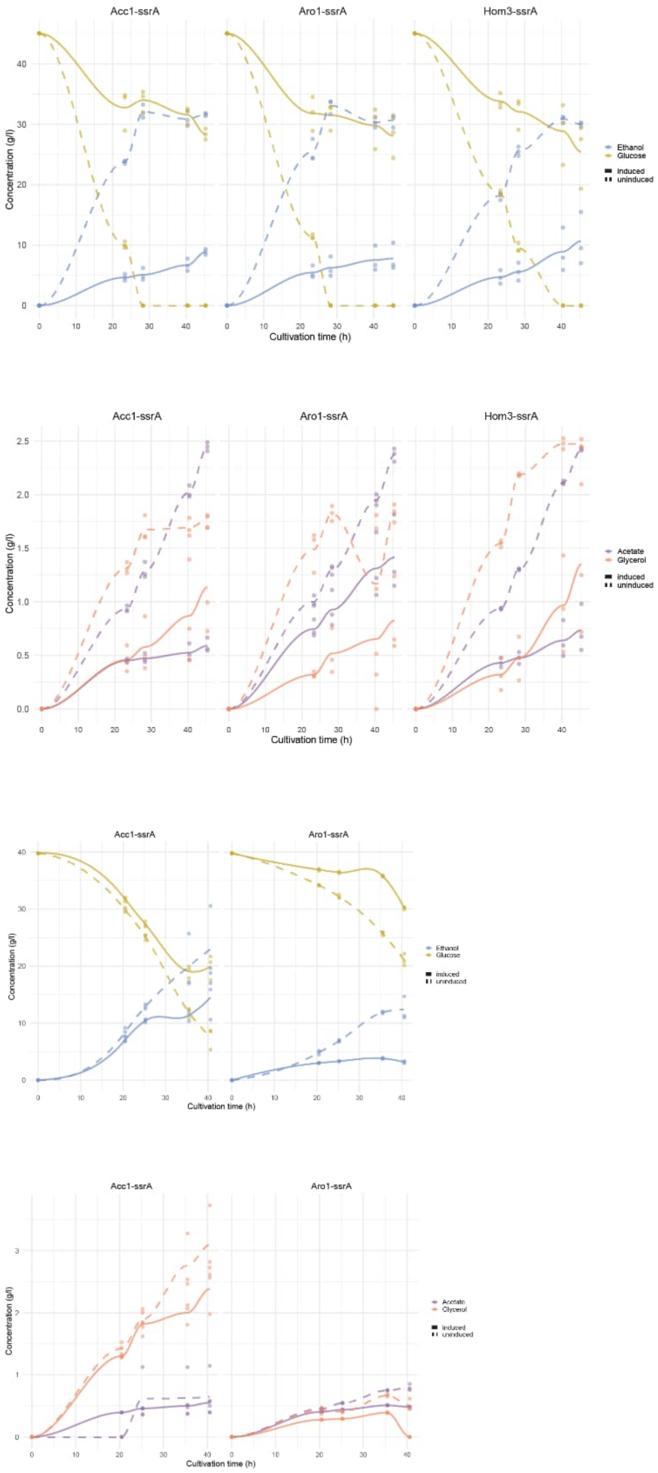

Figure S5. Major byproduct concentrations quantified with HPLC. ClpXP proteasome was induced during exponential growth of strains having either cis,cis-muconic acid pathway (at 13 h) or glycolic acid pathway (at 8 h) and an essential metabolic enzyme ssrA-tagged. Extracellular metabolite concentrations over time are shown for glucose in yellow, ethanol in blue, acetate in purple, and glycerol in orange. Curves of cultures in which the ClpXP proteasome was and was not induced are shown with full line and dashed line, respectively. The curves are shown as loess fits to averages of three biological replicates.

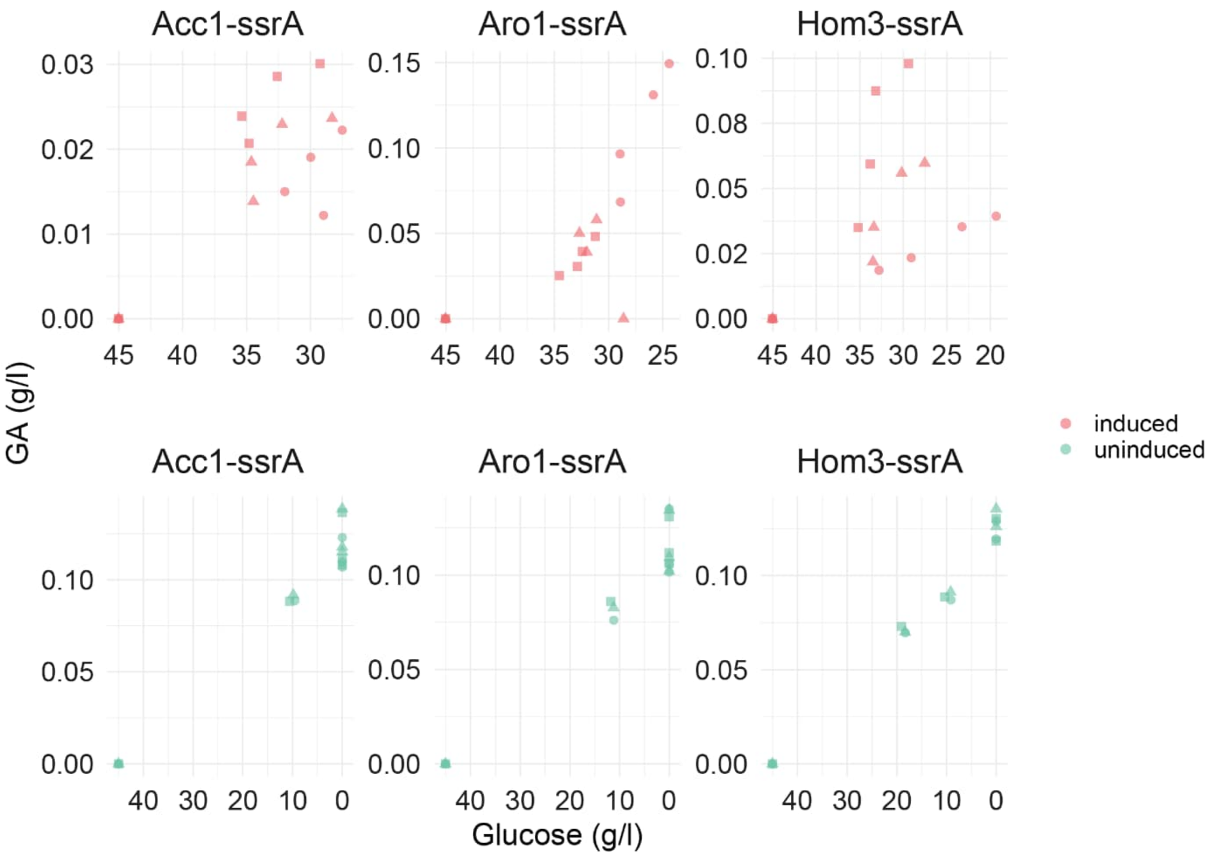

Figure S6. Concentration of glycolic acid as a function of glucose concentration in strains having either Acc1, Aro1 or Hom3 ssrA-tagged, with ClpXP induced cultures show separately from uninduced ones. Glycolic acid concentration in cultures when the ClpXP proteasome was and was not induced are shown in red and green, respectively. The three biological replicate cultures are indicated with unique point shapes (square, triangle, or circle). Each point is an average of two technical replicates.

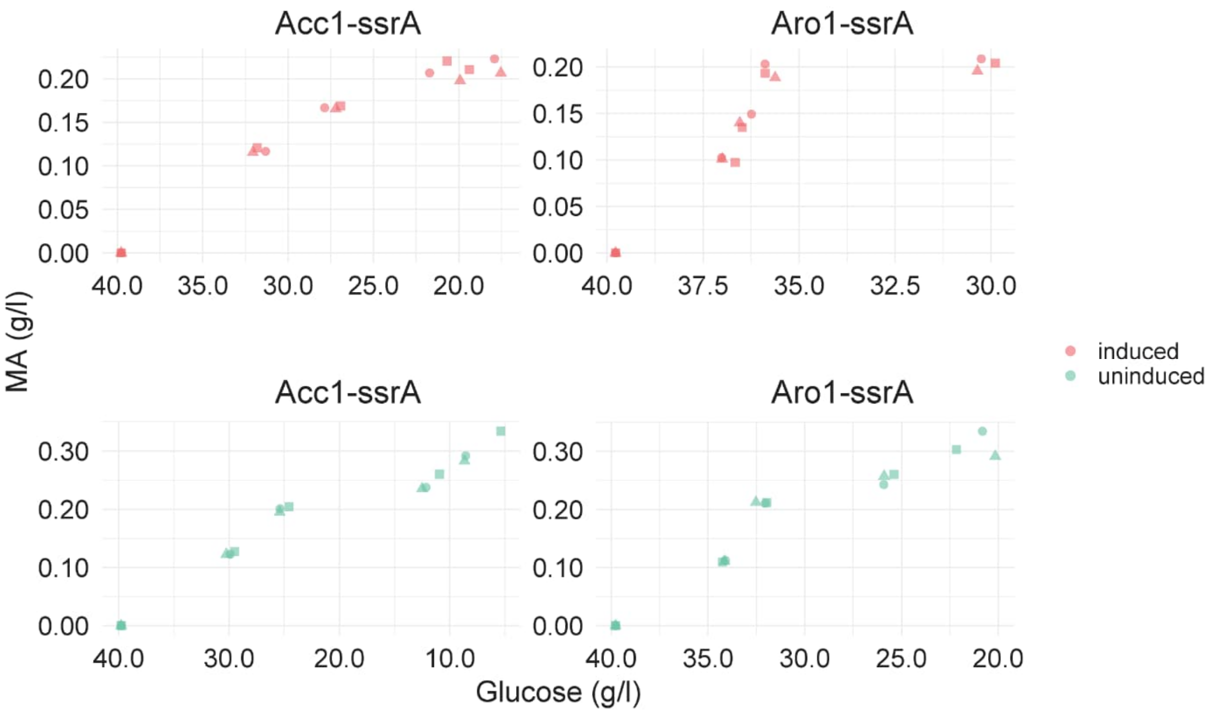

Figure S7. Concentration of cis,cis-muconic acid as a function of glucose concentration in strains having either Acc1 or Aro1 ssrA-tagged, with ClpXP induced cultures show separately from uninduced ones. Cis,cis-muconic acid concentration in cultures when the ClpXP proteasome was and was not induced are shown in red and green, respectively. The three biological replicate cultures are indicated with unique point shapes (square, triangle or circle). Each point is an average of two technical replicates.

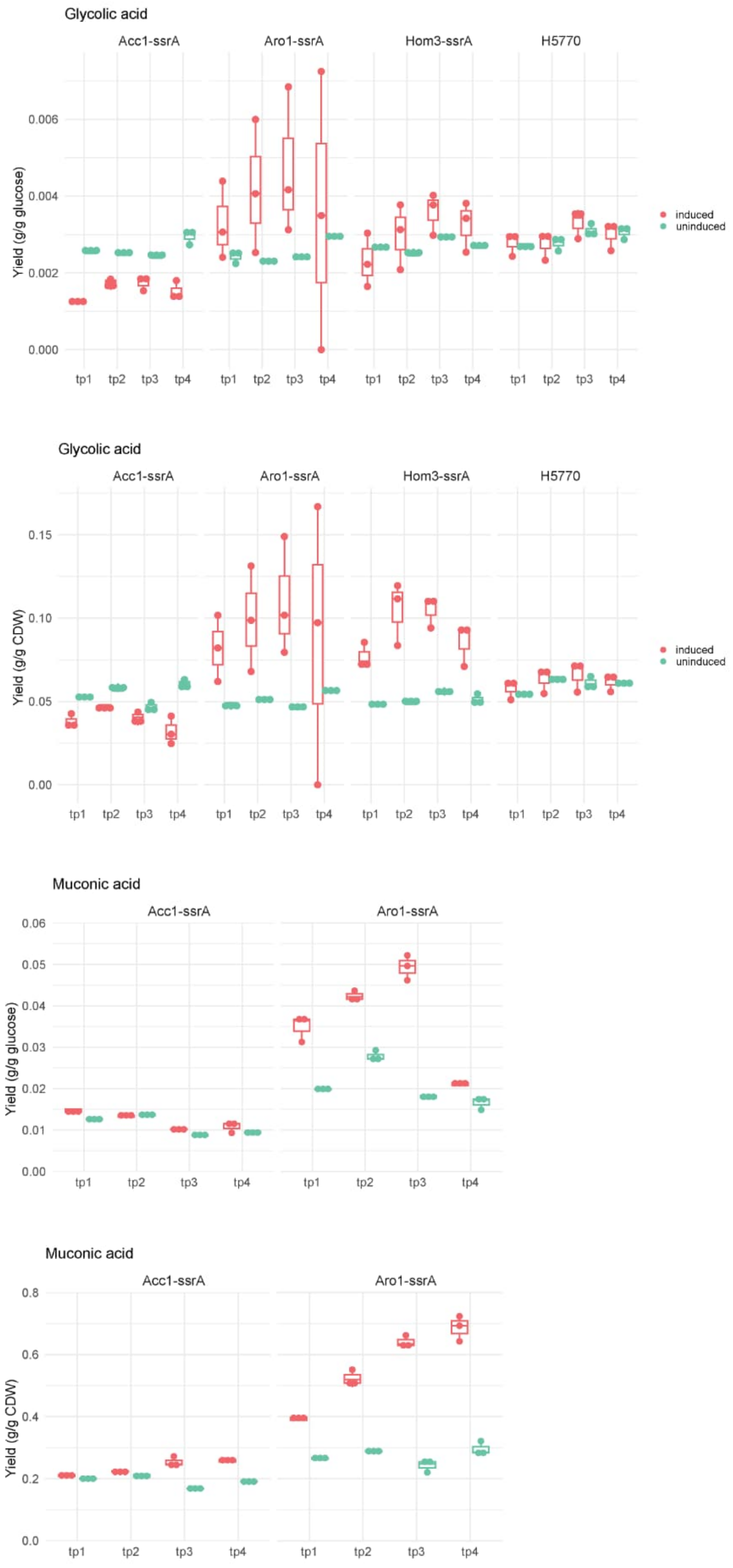

Figure S8. Cis,cis-muconic acid and glycolic acid yields of three biological replicates at several time points (at 21 h, 25 h, 36 h and 41 h for MA, and 23 h, 28 h, 40 h and 45 h for GA) during the incubation of cultures. Cis,cis-muconic acid and glycolic acid yields in cultures when the ClpXP proteasome was and was not induced are shown in red and green, respectively.

Table S3. Plasmids used and cloned in this work.

Code	Description	Reference
p18_6	pGRE-TDH3p-Venus-ssrA	This article
p18_12	pRS406-BID-8BS (TetR)-ClpXP with (PGK1-Bm3R1)/(THI4-Bm3R1)cp	This article (plasmid)<br>Rantasalo et al. 2018 (core promoters/cp)
B7770	pJKLiF-045 Cas9 (KanMX)	Kuivanen et al. 2018
B8947	pCM251	Belli et al. 1998
B8990	pURA-THI4p-TetR'-VP16 (Tet-On)	This article
B9062	pURA-THI4p-TetR*-VP16 (Tet-On, MUT).	This article
B9091	pLEU2-TDH3p-Tet*-on-VP16	This article
B9544	pRP-C-gRNA pSNR52-MssI-tracrRNA-tSUP4 Cas (NAT)	This article
B9974	pRP-C-038 pSNR52p-ACC1-tracrRNA-tSUP4	This article
B9997	pRP-C-038 pSNR52p-ARO1-tracrRNA-tSUP4	This article
B9999	pRP-C-038 pSNR52p-HOM3-tracrRNA-tSUP4	This article
	MoClo plasmids	
B11365	ORF: Pa.AroZ, Parts: 2, 10, ORF, 52, 67, 95	This article
B11366	ORF: Ca.HQD2, Parts: 3, 11, ORF, 53, 72, 95	This article
B11076	ORF: Sc.ARO3K222L, Parts: 2, 12, ORF, 54, 67, 95	This article
B10976	ORF: Sc.ARO4K229L, Parts: 3, 13, ORF, 55, 72, 95	This article
B11512	ORF: Ec.AROB, Parts: 2, 16, ORF, 56, 67, 95	This article
B11513	ORF: Ec.AROD Parts: 3, 14, ORF, 56, 72, 95	This article
B11331	ORF: Kp.AROY, Parts: 2, 9, ORF, 51, 72, 95	This article
B10978	ORF: Sc.PAD1, Parts: 3, 15, ORF, 51, 72, 95	This article
B11131	pYTK096-pHHF2-ARO3K222L-tPGK1-pTEF1-ARO4K229L-tENO2	This article
B11362	pYTK096-pCCW12-Pa.AROZ-tSSA1-pPGK1-Ca.HQD2-tADH1	This article
B13160	pYTK096-pTDH3-Kp.AROY-tENO1-pHHF1-Sc.PAD1-tENO1	This article
B13159	pYTK096-pHTB2-Ec.AROB-tTDH1-pTEF2-Ec.AROD-tTDH1	This article
	USER & EasyClone plasmids	
B12407	ARO3K222L + ARO4K229L + pCfB2899	This article

B12408	Ec.AROB + Ec.AROD + pCfB3034	This article
B12409	Pa.AROZ + Ca.HQD2 + pCfB3035	This article
B12410	Kp.AROY + Sc.PAD1 + pCfB3036	This article
B11962	X-2-PGK1-FAT2	This article
B11964	XI-5-TEF1-OXA-PGK1t	This article
B11963	XII-4-TDH3-panE2	This article
B11795	GLYR1 + pCf2909	This article
B12983	pTDH3-GAPN + pCfB3035	This article
	EasyClone-MarkerFree Integrative Vector	
B9200	X-2 integration plasmid pCfB2899	Jessop-Fabre et al., 2016
B9201	X-3 integration plasmid pCfB3034	Jessop-Fabre et al., 2016
B9202	X-4 integration plasmid pCfB3035	Jessop-Fabre et al., 2016
B9203	XI-1 integration plasmid pCfB3036	Jessop-Fabre et al., 2016
B10447	XI-5 integration plasmid pCfB3037	Jessop-Fabre et al., 2016
B10448	XII-4 integration plasmid pCfB3040	Jessop-Fabre et al., 2016
B10452	XII-5 integration plasmid pCfB2909	Jessop-Fabre et al., 2016
	gRNA	
B8918	X-2 gRNA, pCfB3020	Jessop-Fabre et al., 2016
B8919	X-3 gRNA, pCfB3041	Jessop-Fabre et al., 2016
B8920	X-4 gRNA, pCfB3042	Jessop-Fabre et al., 2016
B8921	XI-I gRNA, pCfB3043	Jessop-Fabre et al., 2016
B8928	XII-5 gRNA, pCfB3050	Jessop-Fabre et al., 2016
B10453	X-2, XI-5 and XII-4 gRNA, pCfB3053	Jessop-Fabre et al., 2016

Rantasalo, A.; Kuivanen, J.; Penttilä, M.; Jäntti, J.; Mojzita, D., Synthetic Toolkit for Complex Genetic Circuit Engineering in *Saccharomyces cerevisiae*. *ACS Synth Biol* 2018, *7* (6), 1573-1587.

Kuivanen, J.; Holmström, S.; Lehtinen, B.; Penttilä, M.; Jäntti, J., A High-Throughput Workflow for CRISPR/Cas9 Mediated Combinatorial Promoter Replacements and Phenotype Characterization in Yeast. *Biotechnology Journal* 2018, *13* (9), 1700593.

Bellí, G.; Garí, E.; Piedrafita, L.; Aldea, M.; Herrero, E., An activator/repressor dual system allows tight tetracycline-regulated gene expression in budding yeast *Nucleic Acids Res.* 1998, *26* (4), 942-947.

Jessop-Fabre, M. M.; Jakočiūnas, T.; Stovicek, V.; Dai, Z.; Jensen, M. K.; Keasling, J. D.; Borodina, I., EasyClone-MarkerFree: A vector toolkit for marker-less integration of genes into *Saccharomyces cerevisiae* via CRISPR-Cas9. *Biotechnology Journal* 2016, *11* (8), 1110-1117.

Table S4. Sequences of the oligos used for annealed donor DNA and gRNA plasmids.

Code	Name	DNA sequence
N1	ACC1	AAACTTCTCCGCAGTGAAAGATAAATGATCCAACAATTTTTCTTTATCATGTTTTAGAGC TAGAAATAGCAAGTTAAAAT
N2	ACC1 rev	ATTTTAACTTGCTATTTCTAGCTCTAAAACATGATAAAGAAAAATTGTTGGATCATTTATC TTTCACTGCGGAGAAGTTT
N3	ARO1	AAACTTCTCCGCAGTGAAAGATAAATGATCTATTGTCTACTCTTTCGTAAGTTTTAGAGCT AGAAATAGCAAGTTAAAAT
N4	ARO1 rev	ATTTTAACTTGCTATTTCTAGCTCTAAAACTTACGAAAGAGTAGACAATAGATCATTTATCT TTCACTGCGGAGAAGTTT
N5	HOM3-1	TGCGCATGTTTCGGCGTTCGAAACTTCTCCGCAGTGAAAGATAAATGATCATTAAAGTGA AGAAGAAAGGGTTTTAGAGCTAGAAATAGCAAGTTAAAATAAGGCTAGTCCGTTATCAA
N6	HOM3-1 rev	GTTGATAACGGACTAGCCTTATTTTAACTTGCTATTTCTAGCTCTAAAACCCTTTCTTCTTCA CTTTAATGATCATTTATCTTTCACTGCGGAGAAGTTTCGAACGCCGAAACATGCGCA
N7	HOM3-2	AAACTTCTCCGCAGTGAAAGATAAATGATCTTAGAACAATTGAAAAGACTGTTTTAGAGC AGAAATAGCAAGTTAAAAT
N8	HOM3-2 rev	ATTTTAACTTGCTATTTCTAGCTCTAAAACAGTCTTTTCAATTGTTCTAAGATCATTTATCTT CACTGCGGAGAAGTTT
N9	ACC1 F	AGCGACCATGACAATGCTATTGATGGATTATCTGAAGTTATCAAGATGTTATCTACAGATG ATAAAGAAAAATTGTTGAAGACTTTGAAAGCAGCCAATGACGAAAATTATGCGTTAGCT
N10	ACC1 R	TATTAGCAACTAAATTAAATTACATCAATACTATTTTTTTTTTTTACTTTACAAGGTTTATGC AGCTAACGCATAATTTTCGTCATTGGCTGCTTTCAAAGTCTTCAACAATTTTTCTTT
N11	ARO1 F	GCTCAGTTTGAAAAGTGGACAGGATTCAAGGGCCCTTTCAAGGCCATTTTTGATGCAGTT ACGAAAGAGGCAGCCAATGACGAAAATTATGCGTTAGCTGCATAGACAATAATATATCTA
N12	ARO1 R	TAGTAAAGATATCATAGAAGCATTGTAAAATATAAAAAAGGATAGATATATTATTGTCTA TGCAGCTAACGCATAATTTTCGTCATTGGCTGCCTCTTTCGTAACTGCATCAAAAATGGC
N13	HOM3-1 F	TCAAACCAATTTGAACATGCCATTGATGAACGTTTAGAACAATTGAAAAGACTTGGAATT GCAGCCAATGACGAAAATTATGCGTTAGCTGCATAAATCTACCTTTCTTCTTCACTTTAA
N14	HOM3-1 R	TATATATATATATATATATAAAGGGAAAATTAATATTCTATCATTAAAGTGAAGAAGAAA GGTAGATTTATGCAGCTAACGCATAATTTTCGTCATTGGCTGCAATTCCAAGTCTTTTCA
N15	HOM3-2 F	ACAAATACTTCAAACCAATTTGAACATGCCATTGATGAACGTTTAGAACAATTGAAAAGA CTTGCAATTGCAGCCAATGACGAAAATTATGCGTTAGCTGCATAAATCCACCTTTCTTCT

N16	HOM3-2 rev	TATATATATAAAGGGAAAATTAATATTCTATCATTAAAGTGAAGAAGAAAGGTGGATTTA GCAGCTAACGCATAATTTTCGTCATTGGCTGCAATTGCAAGTCTTTTCAATTGTTCTAA

Table S5. Primer sequences.

Code	Name	DNA sequence
A1	TetR'-VP16_GA_Fw	ACCAAAAAAATCAACTAACTTTAATTAATGTCTAGATTAGATAAAAGTAAAGTGATTAAC
A2	TetR'-VP16_GA_Rev	GCATTTACTTATAATACAGTTTTGACGCGTCTACCCACCGTACTCGTC
A3	Venus_GA_Fw	AAATTACCGGATCAATTCGGGGGATCTTAATTAAATGTGGTCTCATCCACAATTTGAA
A4	Venus_GA_Rev	AATTACATGATGCGGCCCTCCTGCATTAGGATCCTTTGTACAATTCATC
A5	qPCR_TetR'-VP16_Fw	CTTTACTAAGTCATCGCAATGGAG
A6	qPCR_TetR'-VP16_Rev	TGATCTTCCAATACGCAACC
A19	PGK1cp-ClpP-GA_Fw	CTCGAGTAAGGGGGTGGTTTAGTTTAGTA
A20	PGK1cp-ClpP-GA_Rev	GTCCCTTTCTCCACTGTAGGACATTTTAGGCGCGCCTTTGTTGTAAAAAGTAGATAATTACTTCC
A21	8BS-ENO1cp-ClpX-GA_Fw	ACTAAACTAAACCACCCCCTTACTCGAGCTCGGCTAGCTCTCTATCAC
A22	8BS-ENO1cp-ClpX-GA_Rev	TGAACCGTCCTTTCTCTTGTCAGTCATTATTTTAATTAAGTGTTTGTGTGTTGATAA
A30	ACC1_qPCR_fw	ATAGGCAAGTCGCAACATGG
A31	ACC1_ssrA_qPCR_rev	GTCATTGGCTGCTTTCAAAGTC
A32	ARO1_qPCR_fw	CCTGGATCACAAATGTTAGTACAC
A33	ARO1_ssrA_qPCR_rev	TATTGTCTATGCAGCTAACGC
A34	HOM3_qPCR_fw	TCCTGCGTTATCAATGAATCTG
A35	HOM3_ssrA_qPCR_rev	TGGCTGCAATTCCTAGTCTC
A39	ClpX_GA	CTTACAAAAGATCACGTGATCTGTTGTGCGCGCCTTCAATTAGCTAAGATCATAGC
A40	ClpP_GA	ATTTACTTATAATACAGTTTTGACGCGTTTGGGTTGTCCTCTGAGGACA
A41	PGK1-BMR_GA_Fw	AATACTCCCTATCAGTGATAGAGAGCTAGCCGAGCTGTTTAGTTTAGTAGAACCTCGTG
A42	PGK1-BMR_GA_Rev	TGTCCCTTTCTCCACTGTAGGACATTTTAGGCGCGTTTGTTGTAAAAAGTAGATAATTACTTC
A76	pSNR52p-GA-EcoRI-FW	GGATAACCGTGCGGCCGCCCCTGAATTCTTTGAAAAGATAATGTATGATTATGC
A115	CYC1t_seq_R	CCTAGACTTCAGGTTGTCTAAC
A142	HOM3_ORF_qPCR_fw	CGACCGCTTCATTCTCAATCC
A143	HOM3_ORF_qPCR_re	GAACAAACAACTCAACTTCTCACC

A144	KanMX_qPCR_fw	GAATTTATGCCTCTTCCGACCA
A145	KanMX_qPCR_rev	ATCACTCGCATCAACCAAACC
DM56	pRSET-seq-R	TAGTTATTGCTCAGCGGTGG
DM484	Sc_IPP1_qPCR_F	ACTTTGAACCCAATCATCCA
DM485	Sc_IPP1_qPCR_R	CACCAACTGCCTTAGTTTCTG
DM892	PDC1pc_PacI_R	TTTAATTAATTTGACTGTGTTATTTTGCG
DM1051	8op_F	AAAGGCCACAGTTTCCGGGGAG
DM1154	TDH3p_LexA_GA_F	CCAAGAACTTAGTTTCGAATAAACACAC
DM1221	yomVenus_qPCR_F	CACAATGTTTACATCACTGCTG
DM1222	yomVenus_qPCR_R	CTTTGGATAAGGCAGATTGATAGG
DM1256	ADHt_seq_R	GAGAAAGCAACCTGACCTACAGG
DM1258	CP_gBlock_F	TACTGCTAGTGCTGTATATAAACACAGC
DM1259	CP_gBlock_R	ACTCCTTGATGATGGCCATGTT
DM1284	ClpP_F	GTAATTATCTACTTTTTACAACAAATTAATTAAAATGTCCTACAG
DM1285	ClpP_R	TCATAAATCATAAGAAATTCGCGGATCC
DM1286	ClpX_F	CTGCTTATCAACACACAAACACTTAATTAAAATGACTG
DM1287	ClpX_R	GAATCATGAGTTTTATGTTAATTAGCGTCG
DM1301	sTF+4BS_R	GTAAATGTAAGTTTCACGAGGTTCTAC
DM1304	clpP_qPCR_F	CTACCAAGGACAAGCAACAG
DM1305	clpP_qPCR_R	GATACTATCCACCAAGCCGT
DM1306	clpX_qPCR_F	TAGCCCAAGTTGAACCAGAG
DM1307	clpX_qPCR_R	TCACGAAACTCCAAATCTACACC
DM1419	Sc_TEF1_qPCR_F	AACATGATTGAAGCTACCACC
DM1420	Sc_TEF1_qPCR_R	GCACAGTACCAATACCACCA
DM1478	CEN_GA_R	CAGCTATGACCATGATTACGCC
DM1574	ENOcp_GA_F	CAACTGCTTATCAACACACAAACAC
DM1688	4BS-SrpR_ENO1_rev	GAAATCGTGGAGATCATGTGTGCCAGAAAAGG
DM1913	VP16_PDC5t_qPCR_R2	ATCTATGCCAATTATTTACCTAAACATC
N66	TDH3_prom_FRW	CAA CGG AAT GCG TGC G TCA TTA TCA ATA CTG CCA TT
N67	TDH3_prom_REV	T TTG ACT GTG TTA TTT TGC GTG AGG TT TGT TTG TTT ATG
N68	GapN_FRW	CAT AAA CAA ACA AA CCT CAC GCA AAA TAA CAC AGT CAA A

N69	GapN_REV	AGA GCG GAT GAA TGC ACG CG CAT AAA TCA TAA GAA ATT CG
N70	gapN_seq_FRW	GGGCTCAGAAATAGGCGATT
N71	gapN_seq_REV	ACTGATCGCGATTCCTGTGT
V1	PGK1p_FRW	CAACGGAATGCGTGCGGTGAGTAAGGAAAGAGTGAG
V2	PGK1p_REV	TGTTTTATATTTGTTGTAAAAAGTAG
V3	TDH3p_FRW	CAACGGAATGCGTGCG CAGTTCGAGTTTATCATTATCAAT
V4	TDH3p_REV	TTTGTTTGTTTATGTGTGTTTATTCGA
V5	TEF1p_FRW	CAACGGAATGCGTGCGCCTTGCCAACAGGGAGT
V6	TEF1p_REV	TTTGTAATTAAAACTTAGATTAGATTGC
V7	TEF1-OXA_FRW	CTAATCTAAGTTTTAATTACAAAATGAAGGTTGACACCCCAGAC
V8	PGK1t_REV	AGAGCGGATGAATGCACGCGTTAACGAACGCAGAATTTTCGAGT
V9	FAT2_FRW	CTACTT TTTACAACAAATATAAAACA ATGACAAGTGCCGCTACTGT
V10	FAT2_REV	AGAGCGGATGAATGCACGCG CTACTTATTTCTGCTGCTCTTAGC
V11	panE2_FRW	GAATAAACACACATAAACAAACAAA ATGTCCATTGCTATAGTTGGT
V12	panE2_REV	AGAGCGGATGAATGCACGCG TTAAGCACCTTGAATAGCCAATC
V13	FAT2_Sekv	ATGACAAGTGCCGCTACTGT
V14	panE2_Sekv	ATGTCCATTGCTATAGTTGGT
V15	OXA_Sekv	ATGAAGGTTGACACCCCAGACT
V16	AsisSI3'REV_Sekv	AGAGCGGATGAATGCACGCG
V17	TPIprom_FRW	CAA CGG AAT GCG TGC G GAT CTA CGT ATG GTC ATT TCT TC
V18	GLYR1_REV	AGAGCGGATGAATGCACGCG CTATTCTCTAGAGAATTTAACTGC
V19	GLYR_Sekv1	GTGGTAGATTCGTTGAAGGTC
V20	GLYR_Sekv_REV_1	GACCTTCAACGAATCTACCAC
V21	TPIprom_SEKV1	CAGGTGGTTTGTTACGCATGCT

Table S6. Yeast strains engineered and used in this work.

Name	Composition
H3887	CEN.PK113-7D, parental strain *<br>(URA3, HIS3, LEU2, TRP1, MAL2-8c, SUC2)
H3899	CEN.PK102-3A, parental strain *<br>(ura3-52, HIS3, leu2-3,112, TRP1, MAL2-8c, SUC2)
H3900	CEN.PK102-5B, parental strain *<br>(ura3-52, his3-delta1, leu2-3,112, TRP1, MAL2-8c, SUC2)
H5444	H3900 + pGRE-TDH3p-Venus-ssrA (1 x p18_6)
Y18_51	H5444 + pRS406-8BS-ClpXP (T4/P1-BMR)cp (1 x p18_12)
H5454	Y18_51 + pLEU2-TDH3p-Tet*-on-VP16 (1 x B9091)
Y18_24	H3899 + pLEU2-TDH3p-Tet*-on-VP16 (1 x B9091)
H5495	Y18_24 + pRS406-BID-8BS (TetR)-ClpXP with (PGK1-Bm3R1)/(THI4-Bm3R1)<br>cp (1 x p18_12)
H5498	H5495 + pJKLiF-045 Cas9 (KanMX) (B7770)
H5587	H5498 + ARO1-ssrA (with Cas9)
H5588	H5498 + ACC1-ssrA (with Cas9)
H5589	H5498 + ARO1-ssrA (Cas9 removed)
H5590	H5498 + ACC1-ssrA (Cas9 removed)
H5591	H5498 + HOM3-1-ssrA (Cas9 removed)
H5592	H5498 + HOM3-2-ssrA (Cas9 removed)
H5791	H5587 + AROZ + Ca.HQD2
H5792	H5588 + AROZ + Ca.HQD2
H5793	H5791 + Kp.AROY + PAD1
H5794	H5792 + Kp.AROY + PAD1
H5795	H5793 + Ec.AROB + Ec.AROD
H5796	H5794 + Ec.AROB + Ec.AROD
H5797	H5795 + ARO3K22L + ARO4K229L
H5798	H5796 + ARO3K22L + ARO4K229L

H5799	H5791 (no Cas9)
H5800	H5792 (no Cas9)
H5801	H5793 (no Cas9)
H5802	H5794 (no Cas9)
H5803	H5795 (no Cas9)
H5804	H5796 (no Cas9)
H5805	H5797 (no Cas9)
H5806	H5798 (no Cas9)
H5746	ura3-2 trp1-289 leu2-3,112 his3delta1 MAL2-8C SUC2 FAT2 panE2 OXA
H5770	H5746 + GLYR1
H5772	H5589 + oxalate route (FAT2, OXA, panE2) + GLYR1 (no Cas9)
H5773	H5590 + oxalate route (FAT2, OXA, panE2) + GLYR1 (no Cas9)
H5774	H5592 + oxalate route (FAT2, OXA, panE2) + GLYR1 (no Cas9)
H6120	H5772 + GAPN
H6121	H5773 + GAPN
H6122	H5774 + GAPN

*The parental CEN.PK yeast strains were obtained from Dr. P. Kötter (J.W. Goethe Universität, Germany).

Table S7. Heterologous genes integrated into strains.

Name	Description	Reference
CLPX	*E. coli,* ATpase, ATP-dependant ClpX subunit of the ClpXP protein complex	Farrell et al., 2005
CLPP	*E. coli,* peptidase, proteolytic compartment of the ClpXP protein complex	Farrell et al., 2005
Kp.AROY	*Klebsiella pneumonia*, PCA decarboxylase, GenBank: AB479384	Pyne et al., 2018
Pa.AROZ	*Podospora anserina,* DHS dehydratase, GenBank: XP_001905369	Pyne et al., 2018
PAD1	*S. cerevisiae* strain S288C, FMN prenyltransferase, GenBank: 852150	Pyne et al., 2018
Ca.HQD2	*Candida albicans,* catechol dioxygenase, GenBank: KHC86777	Pyne et al., 2018
Ec.AROB	*E. coli*, DHQ synthase, GenBank: X03867.1	Pyne et al., 2018
Ec.AROD	*E. coli*, DHQ dehydratase, GenBank: X59503.1	Pyne et al., 2018
ARO3K222L	*S. cerevisiae*, *ARO3* with a lysine to leucine exchange at position 222, DAHP synthase isoenzyme	Brückner et al., 2018
ARO4K229L	*S. cerevisiae*, *ARO4* with a lysine to leucine exchange at position 229, feedback-resistant 3-deoxy-D-arabino-heptulosonate-7-phosphate (DAHP) synthase	Brückner et al., 2018
FAT2	*S. cerevisiae* strain S288C, oxalyl-CoA synthetase, GenBank: 852523	Toivari et al., 2019
OXA	*Aspergillus niger,* oxaloacetase	Toivari et al., 2019
panE2	*Methylobacterium extorquens,* oxalyl-CoA reductase	Toivari et al., 2019
GLYR1	*Arabidopsis thaliana,* glyoxylate reductase, GenBank: 822139	Toivari et al., 2019
GAPN	*Triticum aestivum* Q8LK61, NADP-dependent glyceraldehyde-3-phosphate dehydrogenase, GenBank: 543435	This article

Farrell, C. M.; Grossman, A. D.; Sauer, R. T., Cytoplasmic degradation of ssrA-tagged proteins. *Mol. Microbiol.* 2005, *57* (6), 1750-1761.

Pyne, M. E.; Narcross, L.; Melgar, M.; Kevvai, K.; Mookerjee, S.; Leite, G. B.; Martin, V. J. J., An Engineered Aro1 Protein Degradation Approach for Increased *cis,cis*-Muconic Acid Biosynthesis in *Saccharomyces cerevisiae*. *Appl. Environ. Microbiol.* 2018, *84* (17).

Brückner, C.; Oreb, M.; Kunze, G.; Boles, E.; Tripp, J., An expanded enzyme toolbox for production of *cis, cis*-muconic acid and other shikimate pathway derivatives in *Saccharomyces cerevisiae*. *FEMS Yeast Res.* 2018, *18* (2).

Toivari, M.; Ilmén, M.; Penttilä, M. Improved production of oxalyl-coA, glyoxylate and/or glycolic acid. 2019.

Table S8. Table of highly connected metabolites removed from the metabolic network before distance calculations (see Materials and Methods).

s_0419	s_0638	s_1206	s_2859	s_3823	s_0652	s_4004
s_0420	s_0639	s_1207	s_2952	s_3855	s_0653	s_1475
s_0421	s_0687	s_1208	s_2953	s_3880	s_0654	s_1476
s_0423	s_0688	s_1210	s_2966	s_3881	s_0655	s_1477
s_0424	s_0689	s_1211	s_2976	s_3882	s_0656	s_4012
s_0425	s_0690	s_1212	s_2977	s_3883	s_0722	s_4010
s_0426	s_0714	s_1213	s_2994	s_3884	s_0723	s_4113
s_0434	s_0715	s_1214	s_2995	s_3892	s_0724	s_4159
s_0435	s_0716	s_1215	s_3083	s_3906	s_3894	s_1497
s_0437	s_0717	s_1275	s_3093	s_3913	s_3895	s_1498
s_0438	s_0739	s_1276	s_3094	s_3914	s_3896	s_4098
s_0439	s_0740	s_1277	s_3095	s_3917	s_3990	s_4099
s_0456	s_0741	s_1278	s_3108	s_3926	s_3996	s_0394
s_0457	s_0742	s_1279	s_3129	s_3931	s_3887	s_0395
s_0458	s_0782	s_1322	s_3146	s_3935	s_3889	s_0397
s_0460	s_0783	s_1323	s_3147	s_3944	s_3773	s_0398
s_0461	s_0785	s_1324	s_3164	s_3987	s_3774	s_0399
s_0462	s_0786	s_1325	s_3165	s_4011	s_3932	s_0445
s_0467	s_0793	s_1326	s_3215	s_4013	s_4003	s_0446
s_0468	s_0794	s_1329	s_3219	s_4014	s_3879	s_0447
s_0526	s_0795	s_1373	s_3226	s_4016	s_4002	s_0448
s_0528	s_0796	s_1374	s_3228	s_4019	s_3771	s_2784
s_0529	s_0797	s_1437	s_3321	s_4020	s_3893	
s_0530	s_0798	s_1438	s_3324	s_4026	s_3989	
s_0531	s_0799	s_1467	s_3341	s_4027	s_3993	
s_0532	s_0800	s_1468	s_3342	s_4031	s_3994	
s_0533	s_0801	s_1469	s_3359	s_4151	s_3995	
s_0534	s_0802	s_1470	s_3360	s_4160	s_4001	
s_0539	s_0803	s_1538	s_3449	s_4163	s_3912	
s_0541	s_0804	s_1539	s_3657	s_4196	s_3891	
s_0582	s_0805	s_1540	s_3717	s_4197	s_4148	
s_0583	s_0806	s_1545	s_3718	s_4198	s_3832	
s_0584	s_0807	s_1546	s_3719	s_4199	s_3835	
s_0585	s_0808	s_1547	s_3720	s_4200	s_3844	
s_0586	s_0809	s_1548	s_3753	s_4201	s_4029	
s_0587	s_0810	s_1559	s_3766	s_4202	s_4009	
s_0588	s_0837	s_1560	s_3775	s_4203	s_4263	
s_0589	s_0838	s_2783	s_3776	s_4204	s_0846	
s_0590	s_0839	s_2799	s_3777	s_4205	s_0849	
s_0613	s_0840	s_2800	s_3778	s_4206	s_0924	
s_0614	s_0841	s_2808	s_3781	s_2785	s_0925	
s_0615	s_1198	s_2817	s_3782	s_2857	s_0926	
s_0616	s_1199	s_2818	s_3783	s_2834	s_3936	
s_0617	s_1200	s_2820	s_3784	s_2860	s_3536	
s_0618	s_1201	s_2831	s_3786	s_4157	s_3830	
s_0633	s_1202	s_2833	s_3801	s_0647	s_3834	
s_0635	s_1203	s_2840	s_3802	s_0649	s_3842	
s_0636	s_1204	s_2842	s_3813	s_0650	s_4028	
s_0637	s_1205	s_2856	s_3822	s_0651	s_3909	
